# Stereochemistry of *N*-Acyl-5*H*-dibenzo[*b*,*d*]azepin-7(6*H*)-ones

**DOI:** 10.3390/molecules28124734

**Published:** 2023-06-13

**Authors:** Arisa Chiba, Ryoko Tanaka, Mayuno Hotta, Kayo Nakamura, Kosho Makino, Hidetsugu Tabata, Tetsuta Oshitari, Hideaki Natsugari, Hideyo Takahashi

**Affiliations:** 1 Faculty of Pharmaceutical Sciences, Tokyo University of Science, 2641 Yamazaki, Noda-shi, Chiba 278-8510, Japan; 2 Research Institute of Pharmaceutical Sciences, Musashino University, 1-1-20 Shinmachi, Nishitokyo-shi, Tokyo 202-8585, Japan; 3 Faculty of Pharma Sciences, Teikyo University, 2-11-1 Kaga, Itabashi-ku, Tokyo 173-8605, Japan; 4 Graduate School of Pharmaceutical Science, The University of Tokyo, 7-3-1 Hongo, Bunkyo-ku, Tokyo 113-0033, Japan

**Keywords:** axial chirality, atropisomer, dibenzoazepinone, Kv1.3

## Abstract

The stereochemical properties of *N*-acyl-5*H*-dibenzo[*b*,*d*]azepin-7(6*H*)-ones (**2a**–**c**), which inhibit potassium channels in T cells, were examined by freezing their conformational change due to 4-methyl substitution. *N*-Acyl-5*H*-dibenzo[*b*,*d*]azepin-7(6*H*)-ones exist as pairs of enantiomers [(a^1^*R*, a^2^*R*), (a^1^*S*, a^2^*S*)], and each atropisomer is separable at room temperature. An alternate procedure for preparing 5*H*-dibenzo[*b*,*d*]azepin-7(6*H*)-ones involves the intramolecular Friedel–Crafts cyclization of *N*-benzyloxycarbonylated biaryl amino acids. Consequently, the *N*-benzyloxy group was removed during the cyclization reaction to produce 5*H*-dibenzo[*b*,*d*]azepin-7(6*H*)-ones suitable for the subsequent *N*-acylation reaction.

## 1. Introduction

**Scheme 1:**
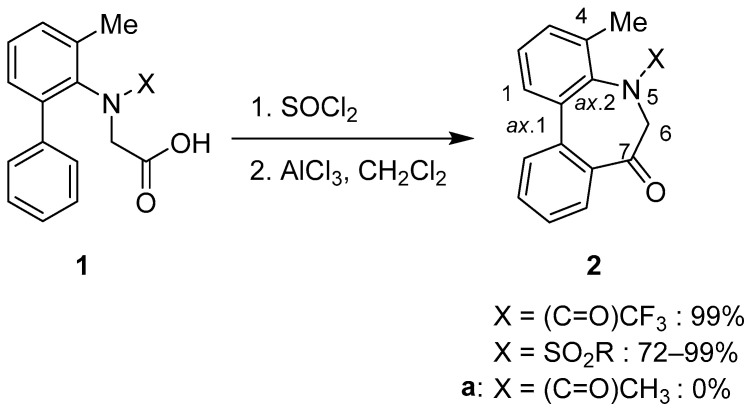
Preparation of *N*-sulfonyl/acyl-5*H*-dibenzo[*b*,*d*]azepin-7(6*H*)-ones.

Recently, our group has become interested in axial chirality and its relation to biological activities. Atropisomers are products of dynamic chirality derived from a restricted rotation around a single bond in a molecule. As axial chirality is caused by a conformational change, it may occur in many organic molecules in various forms. It should be noted that if such molecules have biological activities, axial chirality will be detected by target molecules such as receptors and enzymes. The axial chirality [1,2,3,4,5,6,7,8,9,10] of amides in benzo-fused seven-membered ring nitrogen heterocycles, which are found as the scaffolds of various biologically active molecules, has become the focus of considerable research interest [11,12,13,14,15,16,17]. Although often overlooked, aryl-amides and anilides possess sp^2^–sp^2^ atropisomers based on the Ar–NC(=O) (sp^2^–sp^2^) axis, and target molecules can detect each atropisomer for its biological activity. In the course of our studies aimed at elucidating the relationship between stereochemical property and biological activity in this scaffold [18,19,20,21,22,23,24], we have become interested in the dibenzo[*b*,*d*]azepin-6-one moiety. In 2008, we investigated the stereochemical properties of several derivatives of the γ-secretase inhibitor, LY-411575[25], in which the dibenzo[*b*,*d*]azepin-6-one moiety constitutes an important scaffold. It was elucidated that the two sp^2^–sp^2^ axes, resulting from the Ar–Ar (sp^2^–sp^2^) axis and the Ar–NC(=O) (sp^2^–sp^2^) axis, move in concert to form a stable relative configuration (a pair of enantiomers). In this scaffold, axial chirality with high stereochemical stability enabled kinetically controlled alkylation [26]. Such seven-membered-ring dibenzolactam, dibenzo[*b*,*d*]azepin-6-one, prompted us to study the eight- and nine-membered-ring dibenzolactams by comparing the stereochemical stabilities of the atropisomers and their chemical reactivities toward kinetically controlled stereoselective alkylation [27]. The atropisomers of seven-, eight-, and nine-membered ring dibenzolactams were separated and isolated via chiral HPLC, and their configurations were clarified by an X-ray crystallographic analysis. It was revealed that the two sp^2^–sp^2^ axes, resulting from the Ar–Ar (sp^2^–sp^2^) axis and the Ar–NC(=O) (sp^2^–sp^2^) axis, move in concert in the eight- and nine-membered-ring dibenzolactams. Additionally, an eight-membered-ring benzolactam was found to exist in the most stable configuration owing to the deep, rigid, cage-like ring form, which provides a high barrier to the inversion of the ring system. In this study, we focused on *N*-acyl-/*N*-sulfonyl-5*H*-dibenzo[*b*,*d*]azepin-7(6*H*)-ones **2** (Scheme 1). These compounds exhibit immunosuppressive effects by inhibiting potassium channels (Kv1.3 and IK-1) in T cells [28,29]. Because the Ca^2+^-dependent potassium channel IK-1 and the voltage-gated potassium channel Kv1.3 in human T-cells play pivotal roles during cell proliferation, inhibitors of these channels are promising drug candidates for treating autoimmune diseases such as rheumatoid arthritis and multiple sclerosis [30,31]. In *N*-acyl-5*H*-dibenzo[*b*,*d*]azepin-7(6*H*)-ones **2**, the *E* and *Z* diastereomers around the Ar–NC(=O) bond have been identified, although the presence of chirality has been overlooked. In our previous paper [29], *N*-acyl-5*H*-dibenzo[*b*,*d*]azepin-7(6*H*)-ones **2** were found to exist in the *E*-diastereomer in preference to the *Z*-diastereomer in solution, as also supported by density functional theory calculations (DFT). In addition, stable atropisomers [(a^1^*R*, a^2^*R*), (a^1^*S*, a^2^*S*)] of *N*-acyl-5*H*-dibenzo[*b*,*d*]azepin-7(6*H*)-ones **2** were successfully isolated. Unfortunately, the differences in immunosuppressive effects through the inhibition of potassium channels (Kv1.3 and IK-1) in T cells between the atropisomers of *N*-acyl-5*H*-dibenzo[*b*,*d*]azepin-7(6*H*)-ones **2** were not examined in the previous study. Therefore, we continued the investigation by studying the structure–activity relationship (SAR) of *N*-acyl-5*H*-dibenzo[*b*,*d*]azepin-7(6*H*)-ones **2**. This paper reports an alternative procedure for preparing 5*H*-dibenzo[*b*,*d*]azepin-7(6*H*)-ones, enabling the following *N*-substitution reaction. In addition, the physicochemical properties and inhibitory activities of the potassium channels of the synthesized *N*-acyl-5*H*-dibenzo[*b*,*d*]azepin-7(6*H*)-ones (**2a**–**c**) have been reported.

## 2. Results and Discussion

### 2.1. Preparation of N-Acyl-5H-dibenzo[b,d]azepin-7(6H)-ones

The *N*-Acyl-5*H*-dibenzo[*b*,*d*]azepin-7(6*H*)-one moiety was expected to show latent axial chirality based on the sp^2^–sp^2^ axis resulting from the Ar–Ar (sp^2^–sp^2^) axis (axis 1) and the Ar–NC(=O) (sp^2^–sp^2^) axis (axis 2) (Scheme 1). In our previous paper [29], the conformational change was frozen by introducing a methyl group at C4 to isolate relatively stable stereoisomers. To synthesize the moiety of 4-methyl-substituted 5*H*-dibenzo[*b*,*d*]azepin-7(6*H*)-ones, we carried out the intramolecular Friedel–Crafts acylation of *N*-substituted aryl amino acid derivatives (**1**) (Scheme 1). Acid chlorides prepared in situ from the corresponding *N*-substituted aryl amino acid **1** using thionyl chloride were treated with anhydrous AlCl_3_. Cyclization of *N*-sulfonyl and *N*-trifluoroacetyl derivatives yielded the corresponding 4-methyl-substituted 5*H*-dibenzo[*b*,*d*]azepin-7(6*H*)-one derivatives **2** [29]. However, the reaction of *N*-acetyl derivative **1a** yielded complex mixtures (Scheme 1). Consequently, the electron-withdrawing property of the amino-protecting group in **1** was assumed to be significant to the successful intramolecular Friedel–Crafts acylation of the *N*-substituted aryl amino acids **1**.

**Scheme 2:**
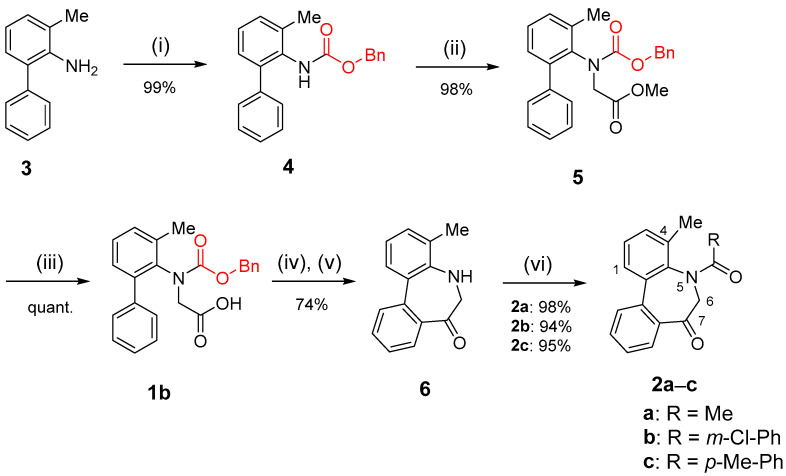
Synthesis of *N*-acyl-5*H*-dibenzo[*b*,*d*]azepin-7(6*H*)-ones. Reagents and Conditions: (i) BnOCOCl, K_2_CO_3_, THF, r.t., 9 days; (ii) BrCH_2_COOMe, NaH, DMF, 0 °C→r.t., 6 h; (iii) NaOH, MeOH, 0 °C→r.t., overnight; (iv) SOCl_2_, reflux, 1 h; (v) AlCl_3_, CH_2_Cl_2_, −78 °C→0 °C, 2 h; (vi) RCOCl, pyridine, THF, r.t., 1–2 h.

In contrast, the intramolecular Friedel–Crafts acylation of *N*-benzyloxycarbonyl (Cbz) compound **1b** (Scheme 2) proceeded efficiently to yield the corresponding 5*H*-dibenzo[*b*,*d*]azepin-7(6*H*)-one **6**. The Cbz group was removed during the cyclization reaction, and the obtained compound **6** was suitable for the following *N*-acylation reaction. As expected, the *N*-acylation of **6** under basic conditions afforded *N*-acyl-5*H*-dibenzo[*b*,*d*]azepin-7(6*H*)-ones **2a**–**c** in good yields. In our previous study [29], 4-unsubstituted *N*-Acetyl-5*H*-dibenzo[*b*,*d*]azepin-7(6*H*)-one was prepared using 4-unsubstituted *N*-trifluoroacetyl-5*H*-dibenzo[*b*,*d*]azepin-7(6*H*)-one via the following two steps: deprotection of the trifluoroacetyl group and *N*-acetylation. However, synthesizing *N*-Acetyl-4-methyl-5,6-dihydro-7*H*-dibenzo[*b*,*d*]azepin-7-one in a similar manner was deemed impossible. We discovered that the deprotection of the trifluoroacetyl group from *N*-trifluoroacetyl-4-methyl-5,6-dihydro-7*H*-dibenzo[*b*,*d*]azepin-7-one could not proceed. Thus, an alternative method for synthesizing various *N*-acyl-5*H*-dibenzo[*b,d*]azepin-7(6*H*)-one derivatives was developed to proceed through the intramolecular Friedel–Crafts acylation of compound **1b**, leading to the formation of 5*H*-dibenzo[*b*,*d*]azepin-7(6*H*)-one **6.** ^1^H, ^13^C NMR spectra of **1b**, **2a**, **2b**, **2c**, **4**, **5**, and **6** are available in Supporting Information.

### 2.2. Stereochemical Properties of N-Acyl-5H-dibenzo[b,d]azepin-7(6H)-ones

**Figure 1:**
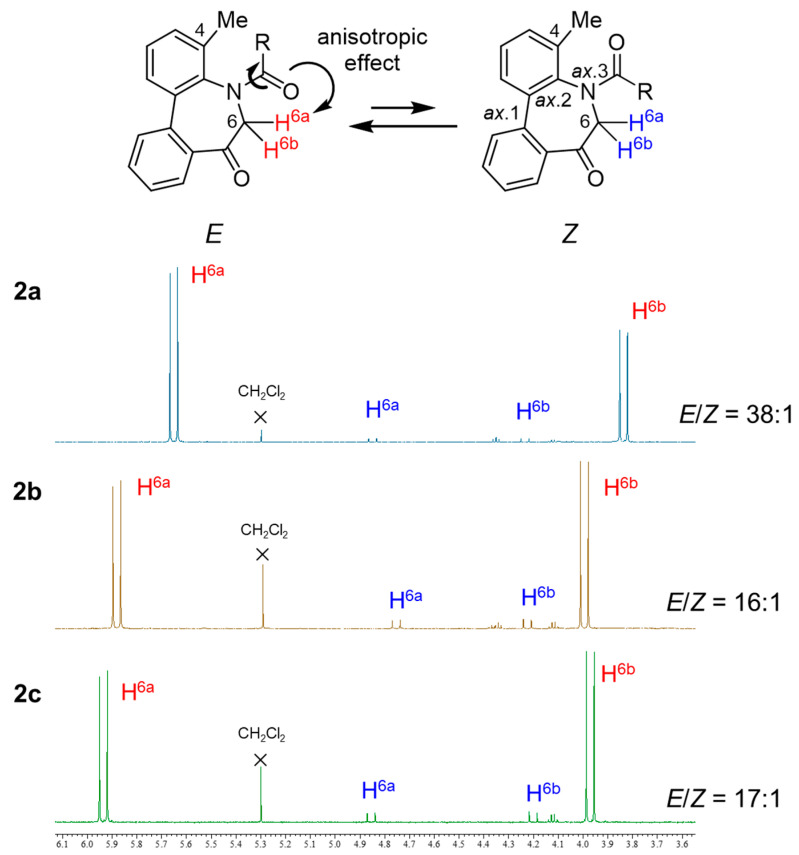
^1^H NMR spectra of *E*/*Z* isomer of *N*-acyl-5*H*-dibenzo[*b*,*d*]azepin-7(6*H*)-ones **2a**–**c**.

*N*-Acyl-5*H*-dibenzo[*b*,*d*]azepin-7(6*H*)-ones (**2a**–**c**) have axial chirality based on the Ar–Ar (sp^2^–sp^2^) axis (axis 1) and the Ar–NC(=O) (sp^2^–sp^2^) axis (axis 2), as well as the *E*/*Z*-amide diastereomers around the N-(C=O) bond (axis 3), as shown in Figure 1. Considering these stereochemical features, dibenzoazepinones might exist as complex stereoisomers. However, our previous studies revealed that the dibenzoazepinone nuclei form stable relative configurations, that is, a pair of enantiomers [(a^1^*R*, a^2^*R*), (a^1^*S*, a^2^*S*)], owing to the concerted rotation of axes 1 and 2 [11,12,13,29]. The relative configuration [(a^1^*R*, a^2^*R*), (a^1^*S*, a^2^*S*)] of *N*-acyl-5*H*-dibenzo[*b,d*]azepin-7(6*H*)-ones was also confirmed via density functional theory (DFT) calculations [29]. Additionally, the stereochemical properties of *N*-acyl-5*H*-dibenzo[*b*,*d*]azepin-7(6*H*)-ones (**2a**–**c**) in a solution were examined using ^1^H NMR spectroscopy. Compounds **2a**–**c** existed as an equilibrium mixture of diastereomers in the solution (CDCl_3_) in ratios of 38:1, 16:1, and 17:1 (Figure 1). In each spectrum, one of the two diastereotopic H-6 protons of a major diastereomer resonates at approximately 5.66 ppm (**2a**), 5.88 ppm (**2b**), 5.94 ppm (**2c**), each 1.82 ppm, 1.88 ppm, and 1.97 ppm downfield from its partner, respectively. A similar downfield shift was observed in our previous study [29] and was ascribed to the anisotropic effect of the carbonyl group. Thus, we considered that **2a**–**c** exist in *E*-amide in preference to *Z*-amide. The exocyclic amide bond (axis 3) did not move in concert with the two endocyclic axes (axes 1 and 2).

**Figure 2:**
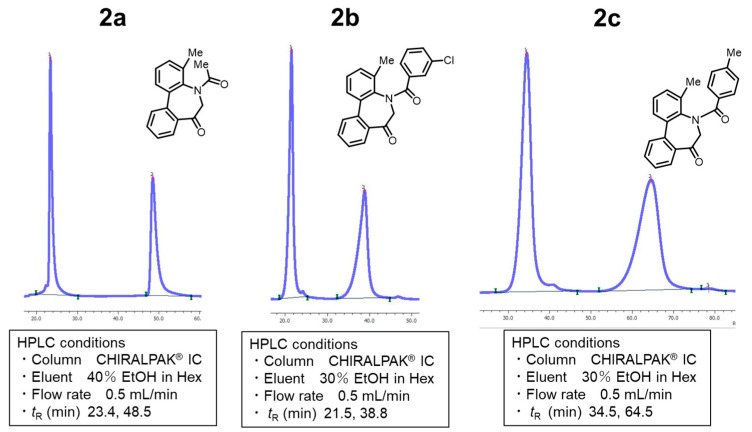
HPLC of *N*-acyl-5*H*-dibenzo[*b*,*d*]azepin-7(6*H*)-ones **2a**–**c**.

Owing to the 4-methyl substituent, the conformational change in **2a**–**c** was fully frozen, and we separated into stable enantiomers [(a^1^*R*, a^2^*R*), (a^1^*S*, a^2^*S*)] using chiral HPLC at room temperature (Figure 2). Therefore, each enantiomer of compounds **2a**–**c** was isolated at room temperature in an enantiomerically pure form.

**Figure 3:**
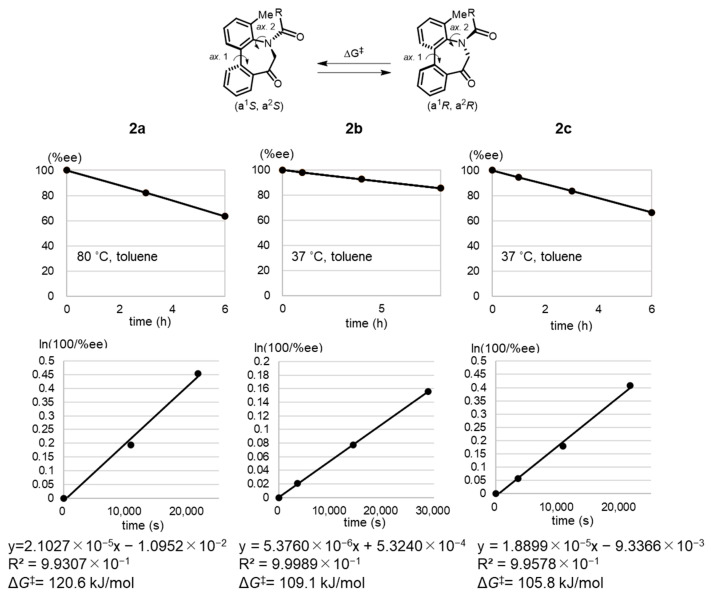
Time-dependent conversion rates (% ee) of **2a**–**c**.

Subsequently, the physicochemical properties of the enantiomerically pure isomers were investigated (Table 1). The Δ*G*^‡^ values of **2a**–**c** were determined based on the time-dependent conversion rate (% ee) (Figure 3) estimated from the chiral HPLC analysis of a toluene solution of each enantiomer. The calculation was conducted according to a procedure reported by Curran [32]. The acetylated derivative **2a** showed the highest energy barrier to rotation (Δ*G*^‡^ = 121 kJ/mol), and the benzoyl derivatives **2b** and **2c** were less stable. Bulky benzoyl substitutions were less effective in reducing conformational changes. Considering that similar results were reported by Graham [33], it is evident that bulkier substituents contribute toward lowering the barrier to rotation. However, determining the specific effects of the *N*-acyl substituent is difficult owing to limited available information on this matter.

### 2.3. Blockage of the Potassium Channel Kv1.3

Finally, the blocking activity of the voltage-gated potassium channel Kv1.3, using 4-aminopyridine as the positive control, was tested for **2c** using patch-cramp technology (Table 2). While the blocking activity of racemate **2c** was not observed at the peak current (open channel inhibition), (−)–**2c** showed more potent affinity than (+)–**2c**. Regarding the activity at the end current (inactivation-dependent inhibition), (−)–**2c** showed more potent affinity than (+)–**2c**, although the enantiomers and racemate exhibited similar levels of affinity (within a 1.6-fold difference).

## 3. Experimental

### 3.1. Chemistry

All the reagents were purchased from commercial suppliers and used as received. Starting materials obtained from commercial suppliers were used without further purification, and starting material **3** was prepared using the previuosly reported method [15]. The reaction mixtures were magnetically stirred, and the reactions were monitored using thin-layer chromatography on pre-coated silica gel plates. Column chromatography was carried out using silica gel (45–60 μm). The extracted solutions were dried over anhydrous Na_2_SO_4_. The solvent was evaporated under reduced pressure. NMR spectra were recorded at 600 MHz for ^1^H NMR and 150 MHz for ^13^C NMR at 296 K. Chemical shifts are provided as parts per million (ppm) downfield of tetramethylsilane, which was used as the internal standard. The coupling constants (*J*) were reported in hertz (Hz). The splitting patterns were abbreviated as singlet (s), doublet (d), triplet (t), quartet (q), multiplet (m), and broad (br) patterns. High-resolution mass spectra (HRMS) were recorded using an electrospray ionization/time-of-flight (ESI/TOF) mass spectrometer. Melting points were recorded using a melting point apparatus and were uncorrected.

#### 3.1.1. Benzyl (3-Methyl-[1,1′-biphenyl]-2-yl)carbamate (**4**)

Benzyl Chloroformate (732 µL, 5.15 mmol) and K_2_CO_3_ (711 mg, 5.15 mmol) were added to a stirred solution of **3** (471 mg, 2.57 mmol) in THF (5.1 mL, 0.5 M) at room temperature under an argon atmosphere. The mixture was stirred at room temperature for 9 d, poured into 2 M HCl aq., and extracted with ethyl acetate. The organic phase was washed twice with a 2 M HCl aq., sat. NaHCO_3_ aq. and brine, subsequently dried and concentrated in vacuum. The residue was purified using column chromatography (silica gel, hexane/diethyl ether = 3:1) to obtain **4** as a white powder (398.5 mg, 99%). mp 51–53 °C; ^1^H NMR (600 MHz, CDCl_3_) δ 7.38–7.21 (m, 12H), 7.17–7.14 (m, 1H), 6.01 (br s, 1H), 5.12 (s, 2H), 2.34 (s, 3H); ^13^C{1H} NMR (150 MHz, CDCl_3_) δ 154.7, 139.8, 139.6, 136.8, 136.6, 132.4, 130.3, 129.1, 128.6, 128.5, 128.3, 128.1, 127.42, 127.35, 67.1, 18.6, several signals overlap. ; IR (ATR) 3264, 1688 cm^−1^; HRMS (ESI-TOF) *m*/*z* calcd for C_21_H_20_NO_2_ 318.1489 (M + H)^+^, found 318.1491.

#### 3.1.2. Methyl *N*-((Benzyloxy)carbonyl)-*N*-(3-methyl-[1,1′-biphenyl]-2-yl)glycinate (**5**)

Sodium hydride (60% in oil) (46.7 mg, 1.17 mmol) was added to a stirred solution of **4** (309 mg, 0.97 mmol) in DMF (4.9 mL, 0.2 M) at 0 °C under an argon atmosphere. After stirring at 0 °C for 20 min, the mixture was treated with methyl bromoacetate (135 µL, 1.46 mmol). After stirring at room temperature for 6 h, the mixture was treated with a 2 M HCl aq. and extracted with ethyl acetate. The extract was washed twice with a 2 M HCl aq., sat. NaHCO_3_ (aq), and brine, were subsequently dried and concentrated. The residue was purified using column chromatography (silica gel, hexane/ethyl acetate = 4:1) to obtain **5** as a yellow powder (372.2 mg, 98%). mp 77–80 °C; ^1^H NMR (600 MHz, CDCl_3_) major: δ 7.39–7.26 (m, 8H), 7.26–7.22 (m, 3H), 7.16–7.12 (m, 2H), 5.29 (d, 1H, *J* = 12.6 Hz), 5.17 (d, 1H, *J* = 12.6 Hz), 3.88 (d, 1H, *J* = 17.4 Hz), 3.60 (s, 3H), 3.23 (d, 1H, *J* = 17.4 Hz), 2.44 (s, 3H), minor: δ 7.39–7.26 (m, 8H), 7.26–7.22 (m, 3H), 7.16–7.12 (m, 2H), 5.30 (d, 1H, *J* = 12.6 Hz), 5.15 (d, 1H, *J* = 12.6 Hz), 3.77 (d, 1H, *J* = 17.4 Hz), 3.52 (s, 3H), 3.28 (d, 1H, *J* = 17.4 Hz), 2.51 (s, 3H); ^13^C{1H} NMR (150 MHz, CDCl_3_) major: δ 169.7, 156.2, 139.6, 138.30, 138.28, 136.3, 130.5, 128.8, 128.62, 128.57, 128.5, 128.32, 128.26, 127.9, 127.6, 68.0, 52.2, 52.0, 18.4, minor: δ 169.9, 156.2, 139.9, 139.8, 139.0, 138.5, 136.7, 130.7, 128.72, 128.65, 128.6, 128.3, 128.2, 128.0, 127.5, 67.8, 52.2, 51.9, 18.3, several signals overlap.; IR (ATR) 1756, 1695 cm^–1^; HRMS (ESI-TOF) *m*/*z* calcd for C_24_H_24_NO_4_ 390.1700 (M + H)^+^, found 390.1702.

#### 3.1.3. *N*-((Benzyloxy)carbonyl)-*N*-(3-methyl-[1,1′-biphenyl]-2-yl)glycine (**1b**)

A total of 10 M NaOH aq. (134 µL, 1.34 mmol) was added to a stirred solution of **5** (104 mg, 0.27 mmol) in MeOH (2.7 mL, 0.1 M) at 0 °C under an argon atmosphere. After stirring overnight at room temperature, the mixture was treated with a 2 M HCl aq. and extracted with ethyl acetate. The extract was washed with 2 M aqueous HCl. with brine, subsequently dried, and concentrated to yield **1b** without further purification. White powder (99.5 mg, quant.). mp 117–120 °C; ^1^H NMR (600 MHz, CDCl_3_) major: δ 7.34–7.27 (m, 8H), 7.25–7.19 (m, 3H), 7.14 (dd, 1H, *J* = 7.8, 1.8 Hz), 7.11 (dd, 1H, *J* = 7.8, 1.8 Hz), 5.27 (d, 1H, *J* = 12.0 Hz), 5.17 (d, 1H, *J* = 12.0 Hz), 3.85 (d, 1H, *J* = 18.0 Hz), 3.30 (d, 1H, *J* = 18.0 Hz), 2.34 (s, 3H), minor: δ 7.34–7.27 (m, 8H), 7.25–7.19 (m, 3H), 7.15–7.13 (m, 1H), 7.12–7.10 (m, 1H), 5.32 (d, 1H, *J* = 12.0 Hz), 5.10 (d, 1H, *J* = 12.0 Hz), 3.76 (d, 1H, *J* = 18.0 Hz), 3.30 (d, 1H, *J* = 18.0 Hz), 2.45 (s, 3H); ^13^C{1H} NMR (150 MHz, CDCl_3_) major: δ 173.8, 156.6, 139.7, 139.4, 138.1, 138.0, 136.1, 130.5, 128.8, 128.64, 128.62, 128.5, 128.3, 128.1, 127.7, 68.3, 52.3, 18.4, minor: δ 174.7, 155.1, 139.9, 139.7, 138.8, 138.5, 136.5, 130.7, 128.8, 128.7, 128.6, 128.4, 128.3, 128.1, 127.6, 67.9, 51.9, 18.3, several signals overlap. ; IR (ATR) 3035, 1738, 1695 cm^–1^; HRMS (ESI-TOF) *m*/*z* calcd for C_23_H_22_NO_4_ 376.1543 (M + H)^+^, found 376.1544.

#### 3.1.4. 4-Methyl-5,6-dihydro-7*H*-dibenzo[*b*,*d*]azepin-7-one (**6**)

Compound **1b** (108 mg, 0.29 mmol) was dissolved in thionyl chloride (573 µL, 0.5 M) under reflux and an argon atmosphere for 1 h. The mixture was subsequently concentrated under reduced pressure. The concentrate was dissolved in dichloromethane (2.9 mL, 0.1 M) at –78 °C under an argon atmosphere, and AlCl_3_ (153 mg, 1.15 mmol) was added. After stirring at 0 °C for 2 h, the mixture was treated with water and extracted using ethyl acetate. The extract was washed with water and brine and subsequently dried and concentrated. The residue was purified using column chromatography (silica gel, hexane/ethyl acetate = 5:1) to obtain **6** as a yellow powder (47.3 mg, 74%). mp 132–135 °C; ^1^H NMR (600 MHz, CDCl_3_) δ 7.91 (ddd, 1H, *J* = 7.8, 1.5, 0.6 Hz), 7.62 (td, 1H, *J* = 7.8, 1.5 Hz), 7.49 (dd, 1H, *J* = 7.8, 1.5 Hz), 7.45–7.41 (m, 2H), 7.23 (dq, 1H, *J* = 7.8, 0.6 Hz), 7.12 (t, 1H, *J* = 7.8 Hz), 4.12 (s, 2H), 3.69 (br s, 1H), 2.36 (s, 3H); ^13^C{1H} NMR (150 MHz, CDCl_3_) δ 204.9, 145.6, 139.3, 137.0, 133.3, 132.9, 131.0, 130.6, 129.2, 128.9, 128.2, 127.6, 124.1, 62.9, 17.8; IR (ATR) 3382, 1664 cm^–1^; HRMS (ESI-TOF) *m*/*z* calcd for C_15_H_14_NO 224.1070 (M + H)^+^, found 224.1072.

#### 3.1.5. 5-Acetyl-4-methyl-5,6-dihydro-7*H*-dibenzo[*b*,*d*]azepin-7-one (**2a**)

Acetyl chloride (54.1 µL, 0.758 mmol) and pyridine (61.2 µL, 0.758 mmol) were added to a stirred solution of **6** (84.6 mg, 0.379 mmol) in tetrahydrofuran (3.8 mL, 0.1 M) at room temperature under an argon atmosphere. The mixture was stirred at room temperature for 2 h, poured into a 2 M HCl aq., and extracted with ethyl acetate. The extract was washed with 1 M NaHCO_3_ aq. and brine, subsequently dried, and concentrated in vacuum. The residue was purified using column chromatography (silica gel, hexane/ethyl acetate = 3:1) to afford **2a** as a white powder (99.0 mg, 98%). mp 136–138 °C; ^1^H NMR (600 MHz, CDCl_3_) *E*-isomer: δ 7.73 (ddd, 1H, *J* = 7.8, 1.8, 0.6 Hz), 7.63 (td, 1H, *J* = 7.8, 1.2 Hz), 7.50 (dd, 1H, *J* = 7.8, 0.6 Hz), 7.47 (td, 1H, *J* = 7.8, 1.2 Hz), 7.43–7.39 (m, 2H), 7.37 (ddd, 1H, *J* = 6.6, 1.8, 0.6 Hz), 5.66 (d, 1H, *J* = 18.0 Hz), 3.84 (d, 1H, *J* = 18.0 Hz), 2.36 (s, 3H), 1.68 (s, 3H), *Z*-isomer: δ 7.88 (dd, 1H, *J* = 7.8, 1.8 Hz), 7.66 (td, 1H, *J* = 7.8, 1.2 Hz), 7.54 (dd, 1H, *J* = 7.8, 1.2 Hz), 7.49–7.46 (m, 1H), 7.44–7.39 (m, 2H), 7.38–7.36 (m, 1H), 4.85 (d, 1H, *J* = 18.0 Hz), 4.24 (d, 1H, *J* = 18.0 Hz), 2.30 (s, 3H), 2.12 (s, 3H); ^13^C{1H} NMR (150 MHz, CDCl_3_) *E*-isomer: δ 203.9, 170.6, 138.9, 138.6, 136.9, 136.7, 135.5, 133.2, 131.6, 129.8, 129.7, 129.2, 129.0, 128.7, 60.0, 21.3, 17.7; IR (ATR) 1666 cm^–1^; HRMS (ESI-TOF) *m*/*z* calcd for C_17_H_16_NO_2_ 266.1176 (M + H)^+^, found 266.1179. Separation of atropisomers. CHIRALPAK^®^ IC (4.6 mmϕ×25 cm), eluent: 30% ethanol in hexane, flow rate: 0.5 mL/min, temperature: 25 °C, detection: 254 nm; former peak, retention time = 23.4 min; latter peak, retention time = 48.5 min.

#### 3.1.6. 5-(3′-Chlorobenzoyl)-4-methyl-5,6-dihydro-7*H*-dibenzo[*b*,*d*]azepin-7-one (**2b**)

Compound **2b** was prepared following the standard procedure described for **2a**. White powder (88.9 mg, 94%). mp 173–176 °C; ^1^H NMR (600 MHz, CDCl_3_) *E*-isomer: δ 7.81 (ddd, 1H, *J* = 7.8, 1.2, 0.6 Hz), 7.73 (td, 1H, *J* = 7.8, 1.2 Hz), 7.59 (dd, 1H, *J* = 7.8, 0.6 Hz), 7.54 (td, 1H, *J* = 7.8, 1.2 Hz), 7.38 (dd, 1H, *J* = 7.2, 0.6 Hz), 7.33 (t, 1H, *J* = 7.2 Hz), 7.20 (ddd, 1H, *J* = 7.8, 2.1, 1.5 Hz), 7.13 (dq, 1H, *J* = 7.2, 0.6 Hz), 7.11–7.10 (m, 1H), 7.05 (td, 1H, *J* = 7.8, 0.3 Hz), 7.02 (dt, 1H, *J* = 7.8, 1.5 Hz), 5.88 (d, 1H, *J* = 18.6 Hz), 4.00 (d, 1H, *J* = 18.6 Hz), 2.10 (s, 3H), *Z*-isomer: 7.78 (dd, 1H, *J* = 7.8, 1.2 Hz), 7.75–7.71 (m, 1H), 7.61–7.58 (m, 1H), 7.55–7.52 (m, 1H), 7.39–7.37 (m, 1H), 7.32 (t, 1H, *J* = 7.2 Hz), 7.18–7.17 (m, 1H), 7.14–7.12 (m, 1H), 7.11–7.10 (m, 1H), 7.06–7.03 (m, 1H), 7.03–7.01 (m, 1H), 4.75 (d, 1H, *J* = 18.6 Hz), 4.23 (d, 1H, *J* = 18.6 Hz), 2.43 (s, 3H); ^13^C{1H} NMR (150 MHz, CDCl_3_) *E*-isomer: δ 203.2, 168.4, 138.64, 138.59, 137.5, 137.1, 136.4, 134.9, 133.7, 133.6, 131.8, 130.6, 129.8, 129.7, 129.4, 129.09, 129.07, 128.6, 128.2, 125.8, 60.7, 17.9; IR (ATR) 1690, 1653 cm^–1^, HRMS (ESI-TOF) *m*/*z* calcd for C_22_H_17_NO_2_Cl 362.0942 (M + H)^+^, found 362.0943. Separation of atropisomers. CHIRALPAK^®^ IC (4.6 mmϕ× 25 cm), eluent: 30% ethanol in hexane, flow rate: 0.5 mL/min, temperature: 25 °C; detection: 254 nm; former peak, retention time = 21.5 min; latter peak, retention time = 38.8 min.

#### 3.1.7. 4-Methyl-5-(4′-methylbenzoyl)-5,6-dihydro-7*H*-dibenzo[*b*,*d*]azepin-7-one (**2c**)

Compound **2c** was prepared following the standard procedure described for **2a**. White powder (285.2 mg, 95%). mp 174–176 °C; ^1^H NMR (600 MHz, CDCl_3_) *E*-isomer: δ 7.80 (ddd, 1H, *J* = 7.8, 1.2, 0.6 Hz), 7.70 (td, 1H, *J* = 7.8, 1.2 Hz), 7.57 (dd, 1H, *J* = 7.8, 0.6 Hz), 7.52 (td, 1H, *J* = 7.8, 1.2 Hz), 7.37 (dd, 1H, *J* = 7.8, 1.2 Hz), 7.31 (t, 1H, *J* = 7.8 Hz), 7.11 (dq, 1H, *J* = 7.8, 1.2 Hz), 7.05 (dt, 2H, *J* = 8.4, 1.2 Hz), 6.90 (dd, 2H, *J* = 8.4, 1.2 Hz), 5.94 (d, 1H, *J* = 19.2 Hz), 3.97 (d, 1H, *J* = 19.2 Hz), 2.22 (s, 3H), 2.06 (s, 3H), *Z*-isomer: δ 7.75 (ddd, 1H, *J* = 7.8, 1.2, 0.3 Hz), 7.71–7.68 (m, 1H), 7.60 (dd, 1H, *J* = 7.8, 1.2 Hz), 7.52–7.49 (m, 1H), 7.38–7.37 (m, 1H), 7.32–7.29 (m, 1H), 7.09 (d, 1H, *J* = 7.8 Hz) 7.05–7.03 (m, 2H), 6.91–6.89 (m, 2H), 4.86 (d, 1H, *J* = 19.2 Hz), 4.20 (d, 1H, *J* = 19.2 Hz), 2.43 (s, 3H), 2.36 (s, 3H); ^13^C{1H} NMR (150 MHz, CDCl_3_) *E*-isomer: δ 203.8, 169.8, 140.8, 139.4, 138.6, 137.8, 137.3, 135.1, 133.4, 131.9, 131.7, 129.7, 129.4, 129.3, 128.9, 128.41, 128.36, 128.1, 60.8, 21.5, 18.0; IR (ATR) 1680, 1645 cm^–1^; HRMS (ESI-TOF) *m*/*z* calcd for C_23_H_20_NO_2_ 342.1489 (M + H)^+^, found 342.1490. Separation of atropisomers. CHIRALPAK^®^ IC (4.6 mm ϕ× 25 cm), eluent: 30% ethanol in hexane, flow rate: 0.5 mL/min, temperature: 25 °C; detection: 254 nm; former peak, retention time = 34.5 min; latter peak, retention time = 64.5 min.

### 3.2. Measurement of the Blocking Activity on the Voltage-Gated Potassium Channel Kv1.3

The assays were performed under the following conditions. The measured parameter was the maximum outward current evoked by stepping to 0 mV from the holding potential. The peak current amplitude was calculated before and after compound addition, and the amount of blocking was assessed by dividing the test compound current amplitude by the control current amplitude. The test compounds were the mean hKv1.3 current amplitudes, collected in the presence of the test compound at each concentration, and the control was the mean hKv1.3 current amplitude, collected for the last 15 s of the control. All the data were filtered for seal quality, seal drop, and current amplitude.

## 4. Conclusions

*N*-acyl-5*H*-dibenzo[*b*,*d*]azepin-7(6*H*)-ones were prepared via the simple intramolecular Friedel–Crafts acylation of *N*-bezyloxycarbonylated biaryl amino acid **1b**. Although the mechanism of the benzyloxycarbonyl group removal during the intramolecular cyclyzation reaction was not clarified, it should be noted that 4-substituted 5*H*-dibenzo[*b*,*d*]azepin-7(6*H*)-one was obtained directly from the *N*-bezyloxycarbonylated biaryl amino acid **1b**. The stereochemistry of the three derivatives **2a**–**c** was elucidated using ^1^H NMR spectroscopy. *E*/*Z* isomers derived from *ax*.3 were detected using ^1^H NMR, and *E* isomers were observed to predominate. Additionally, introducing a Me group at the peri-position reduced the rotation of axes 1 and 2, rendering the enantiomers separable at room temperature. The preliminary results on the difference between the atropisomers for the inhibitory activity of the potassium channel Kv1.3 may be helpful for future drug design. A more detailed investigation of the intramolecular Friedel–Crafts acylation of *N*-bezyloxycarbonylated biaryl amino acid **1b** is under consideration.

## Figures and Tables

**Table 1:**
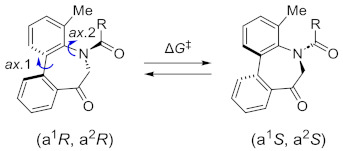
Physicochemical properties of the separated atropisomers of **2a**–**c**.

	R	[α]_D_^a^	Δ*G*^‡^ (kJ/mol) ^b^
**2a**	Me	+42.2 (>99% ee)	121
		−42.1 (>99% ee)	
**2b**	*m*-Cl-Ph	+137.4 (>99% ee)	109
		−134.8 (>99% ee)	
**2c**	*p*-Me-Ph	+135.6 (>99% ee)	106
		−156.6 (>99% ee)	

^a^ Measured in MeOH at 20 °C. ^b^ Racemized in toluene at 80 °C for **2a** and at 37 °C for **2b** and **2c**.

**Table 2:** K_v_ 1.3 blocking activity of **2c** (racemate and atropisomers).

	Estimated IC_50_ (µM)	
	Peak	End
*rac*-**2c**	>30	6.70
(−)-**2c**	20.2	3.88
(+)-**2c**	>30	6.18
4-aminopyridine	767	371

## Data Availability

Data is contained within the article or supplementary material.

## Supplementary Materials

The following supporting information can be downloaded at: https://www.mdpi.com/article/10.3390/molecules28124734/s1, Supporting Information. ^1^H, ^13^C NMR spectra of **1b**, **2a**, **2b**, **2c**, **4**, **5**, and **6**.
