# Supplementary material for: Stereochemistry of N-Acyl-5H-dibenzo[b,d]azepin-7(6H)-ones
Source: Molecules. 2023 Jun 13;28(12):4734. doi: 10.3390/molecules28124734 (PMC10303827; doi:10.3390/molecules28124734)

Supporting Information

Stereochemistry of *N*-acyl-5*H*-dibenzo[*b*,*d*]azepin-7(6*H*)-ones

Arisa Chiba [1], Ryoko Tanaka [1], Mayuno Hotta [1], Kayo Nakamura [1], Kosho Makino [2], Hidetsugu Tabata [3], Tetsuta Oshitari [3], Hideaki Natsugari [4] and Hideyo Takahashi [1,*]

[1] Faculty of Pharmaceutical Sciences, Tokyo University of Science, 2641 Yamazaki, Noda-shi, Chiba 278-8510, Japan

[2] Research Institute of Pharmaceutical Sciences, Musashino University, 1-1-20 Shinmachi, Nishitokyo-shi, Tokyo 202-8585, Japan

[3] Faculty of Pharma Sciences, Teikyo University, 2-11-1 Kaga, Itabashi-ku, Tokyo 173-8605, Japan

[4] Graduate School of Pharmaceutical Science, The University of Tokyo, 7-3-1 Hongo, Bunkyo-ku, Tokyo 113-0033, Japan

* Correspondence: hide-tak@rs.tus.ac.jp; Tel.: +81-47-121-4436

Contents

1. ^{1}H and ^{13}C NMR Spectra…………………………………………………………….. S1

1. ^{1}H and ^{13}C NMR Spectra

^{1}H NMR (600 MHz, $CDCl_3$, ppm) of **4**

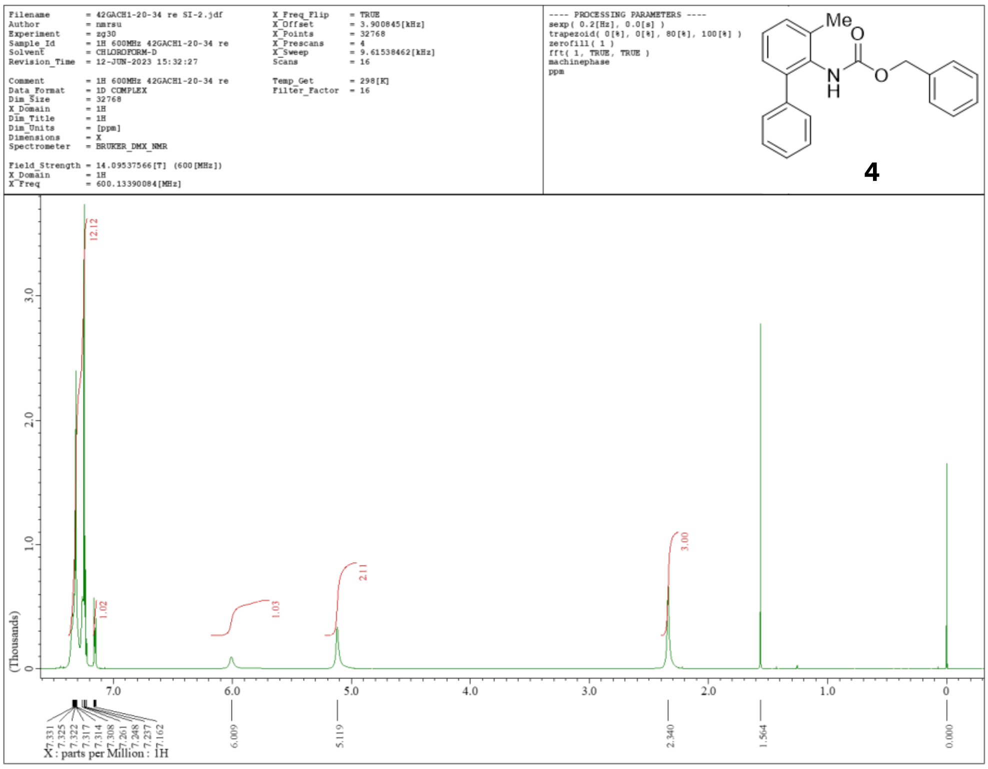

^{13}C NMR (150 MHz, $CDCl_3$, ppm) of **4**

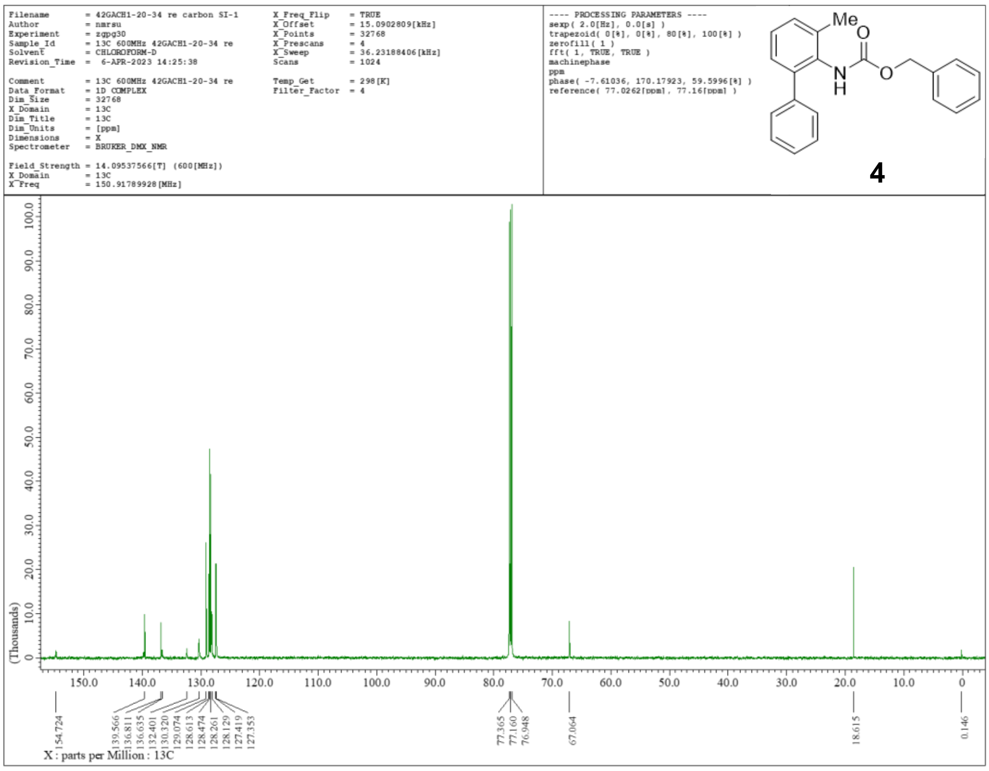

^{1}H NMR (600 MHz, CDCl$_3$, ppm) of **5**

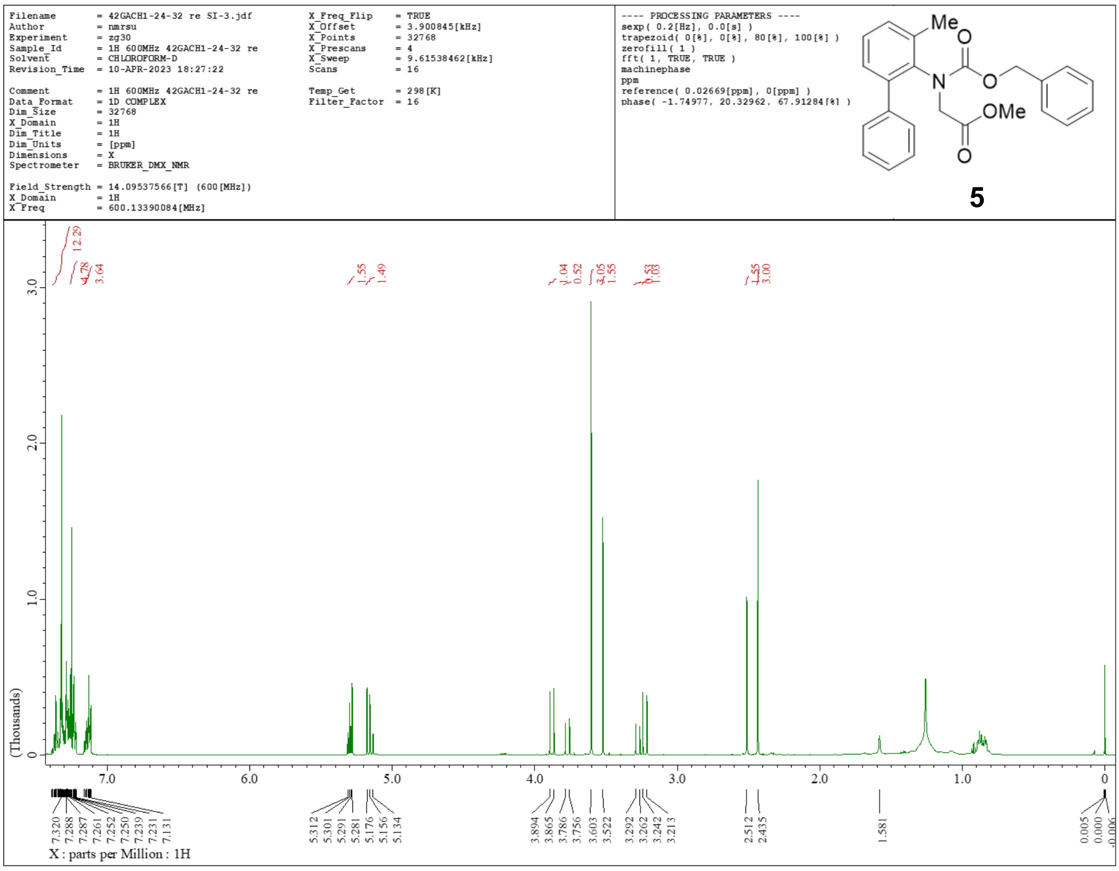

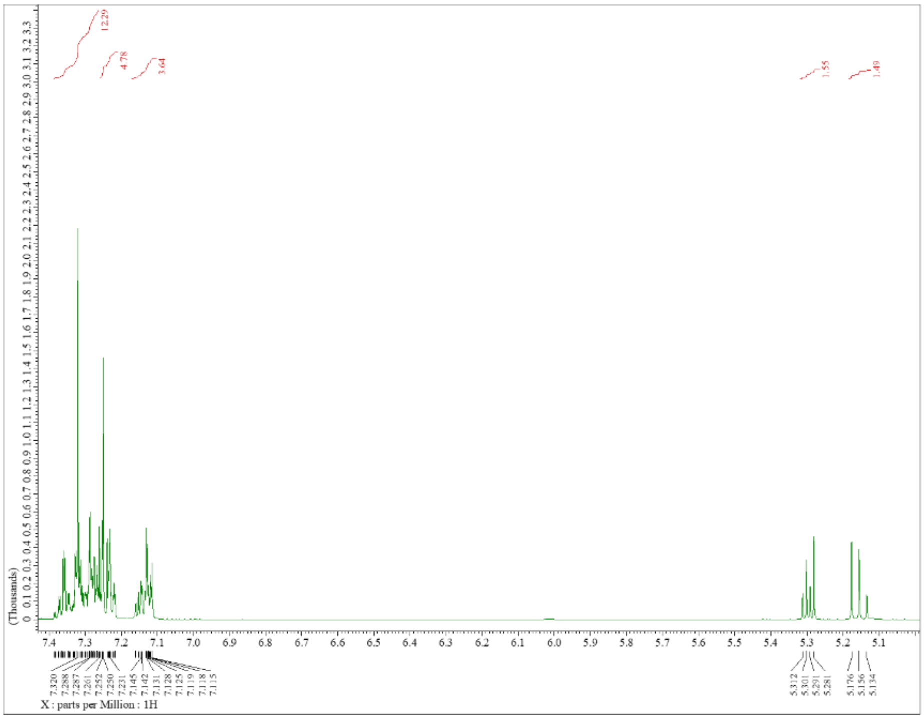

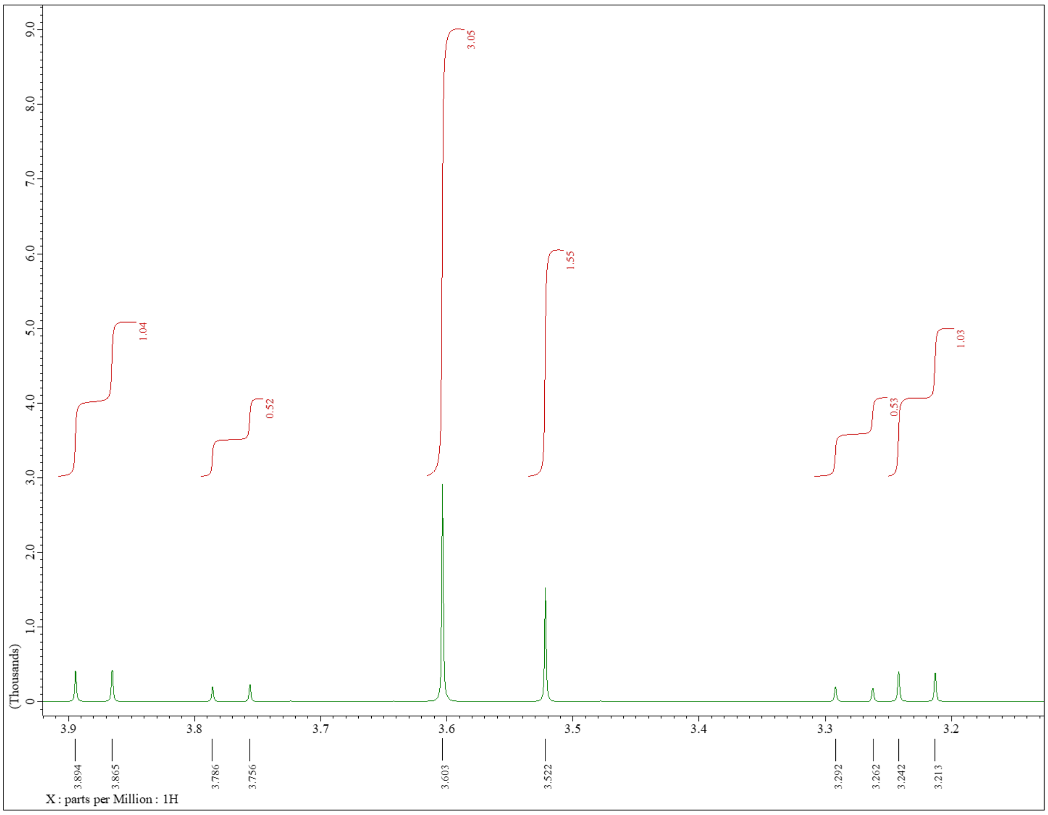
(Thousands)
X : parts per Million : 1H
1.04
0.52
3.05
1.55
0.53
1.03
3.894
3.865
3.786
3.756
3.603
3.522
3.292
3.262
3.242
3.213

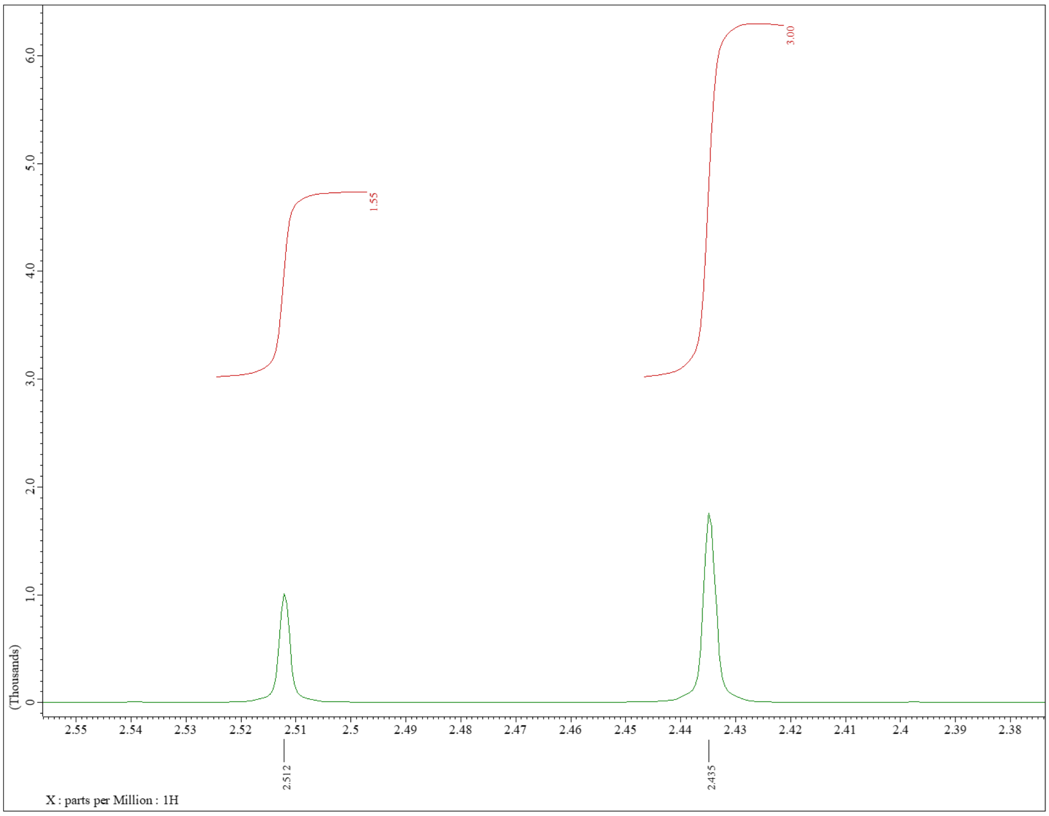
(Thousands)
X : parts per Million : 1H
1.55
3.00
2.512
2.435

^{13}C NMR (150 MHz, $CDCl_3$, ppm) of **5**

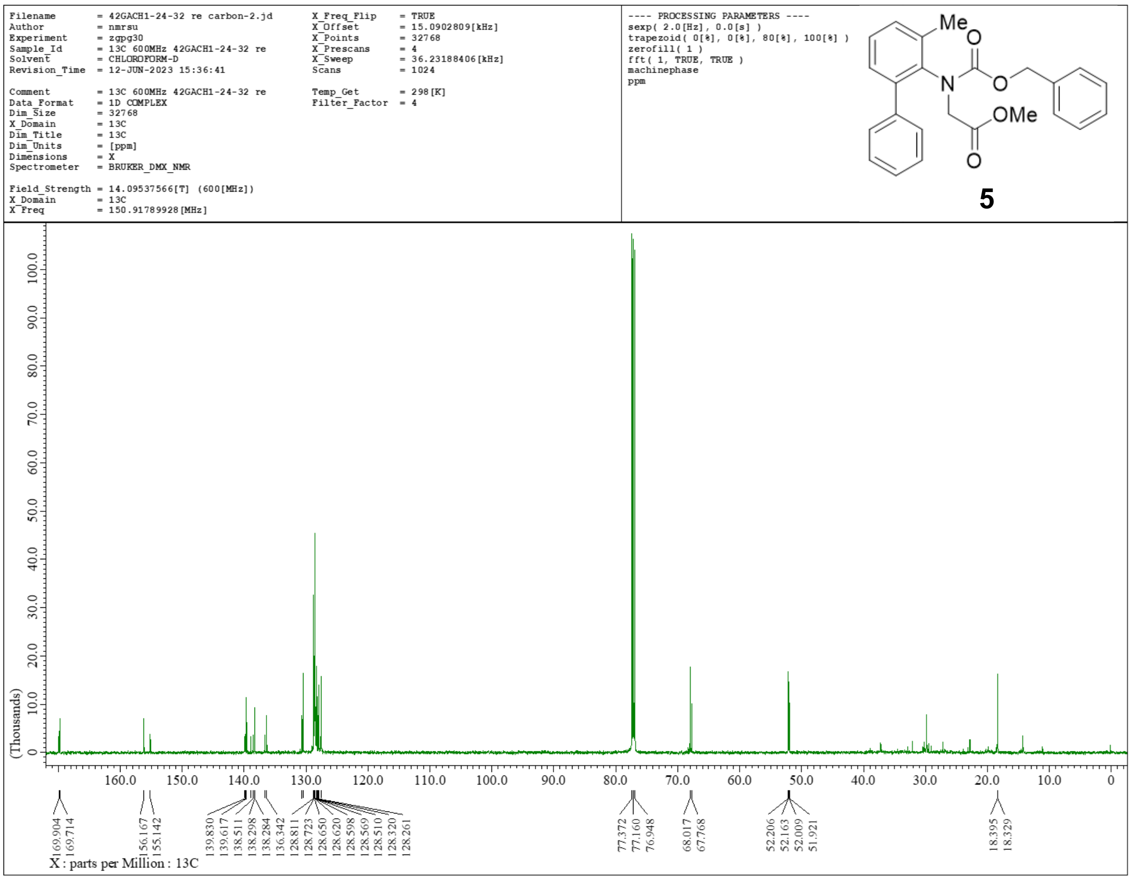

^{1}H NMR (600 MHz, $CDCl_3$, ppm) of **1b**

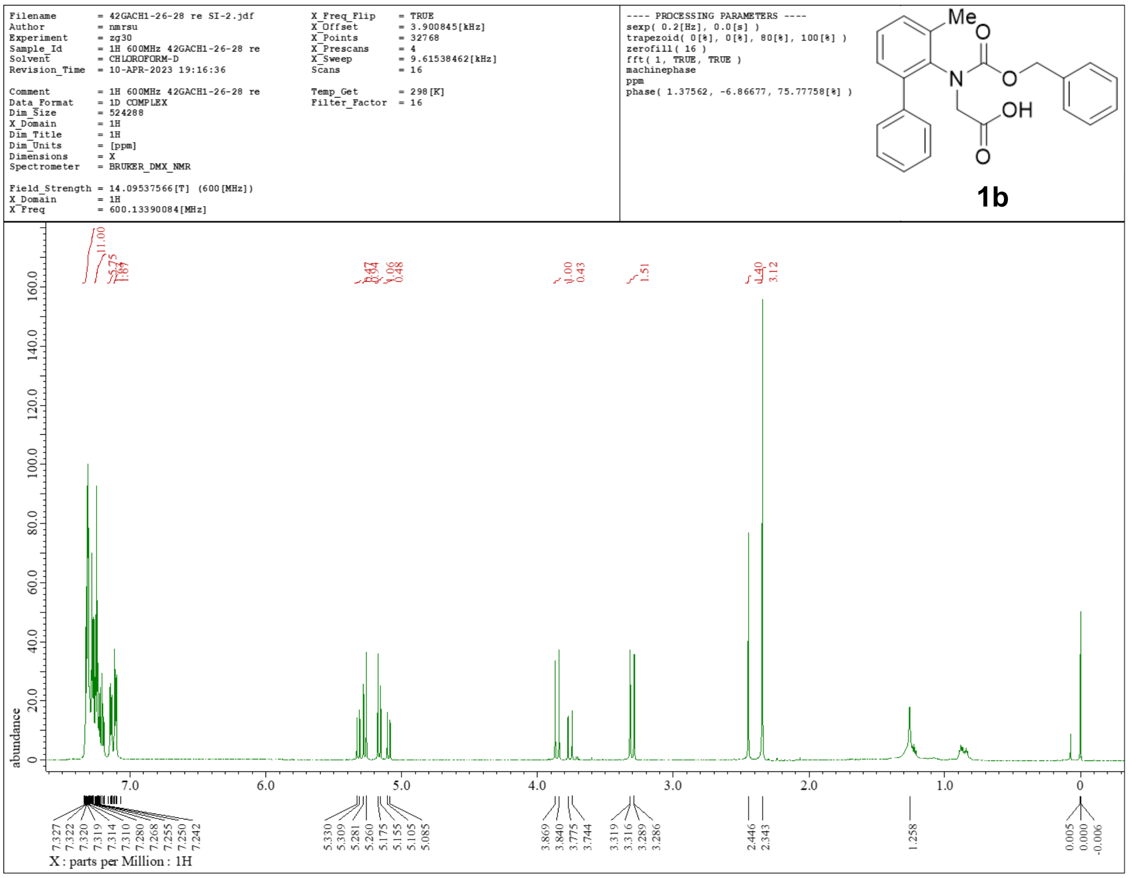

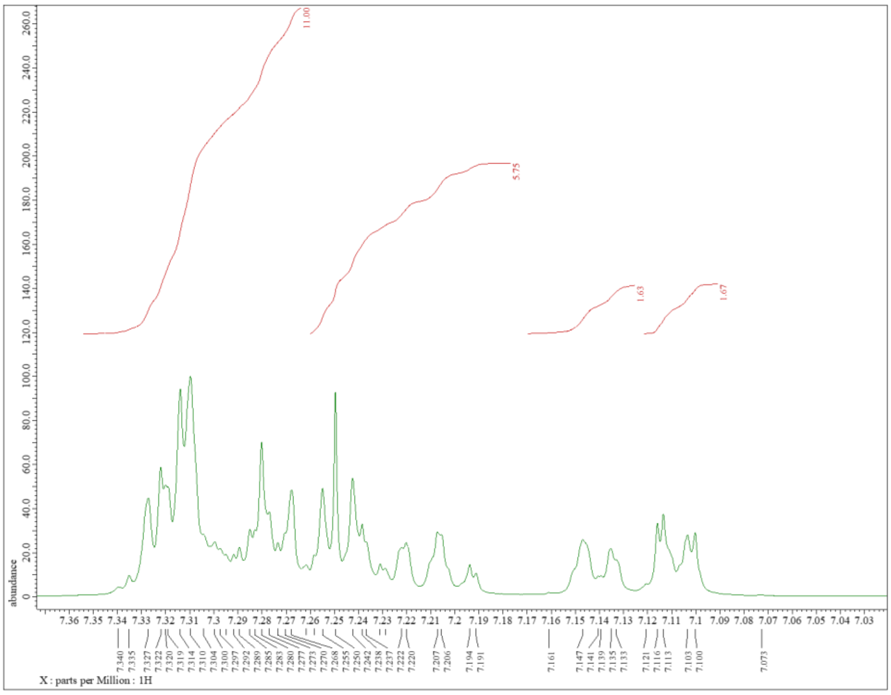

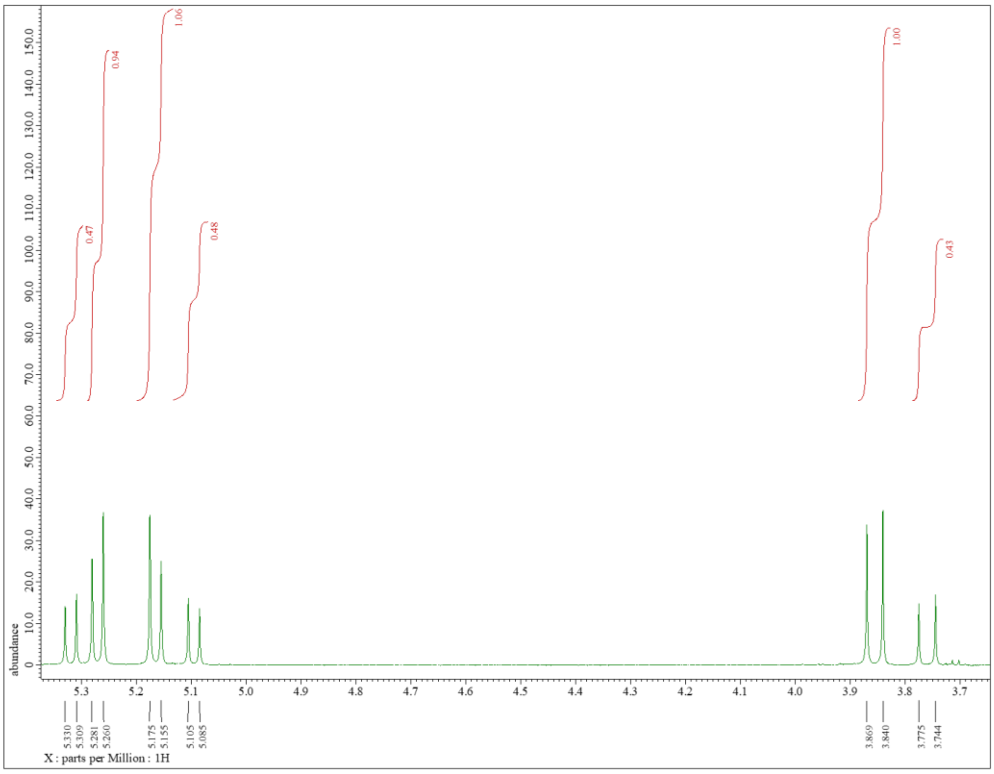
abundance
X : parts per Million : 1H
0.47
0.94
1.06
0.48
1.00
0.43
5.330
5.309
5.281
5.260
5.175
5.155
5.105
5.085
3.869
3.840
3.775
3.744

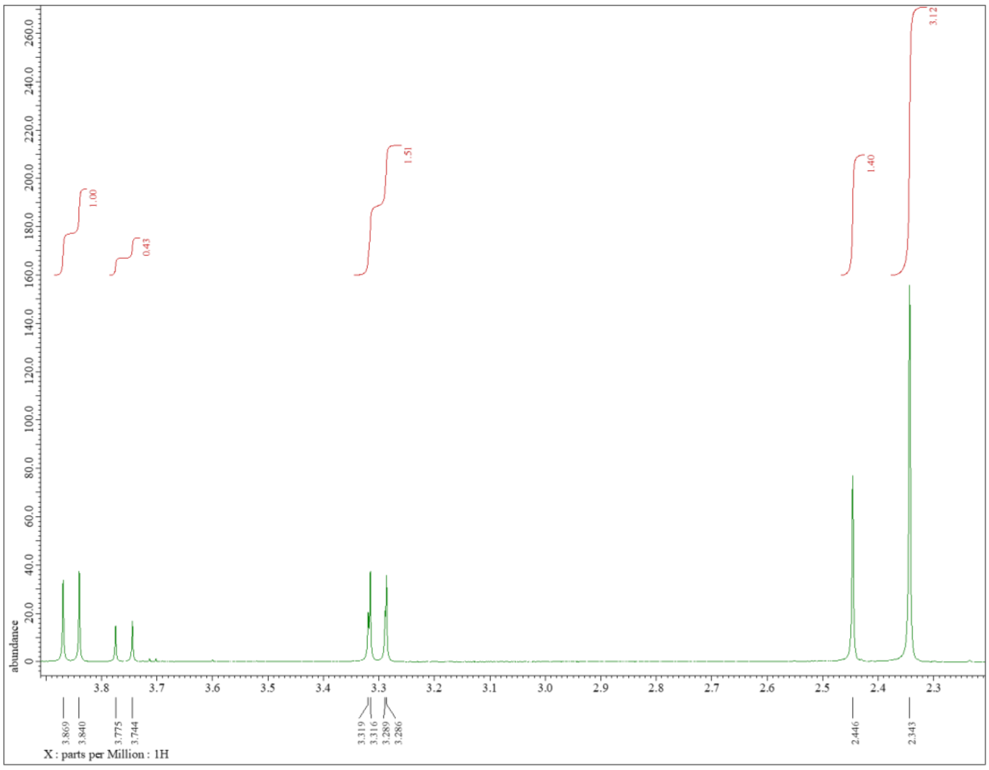
abundance
X : parts per Million : 1H
1.00
0.43
1.51
1.40
3.12
3.869
3.840
3.775
3.744
3.319
3.316
3.289
3.286
2.446
2.343

^{13}C NMR (150 MHz, $CDCl_3$, ppm) of **1b**

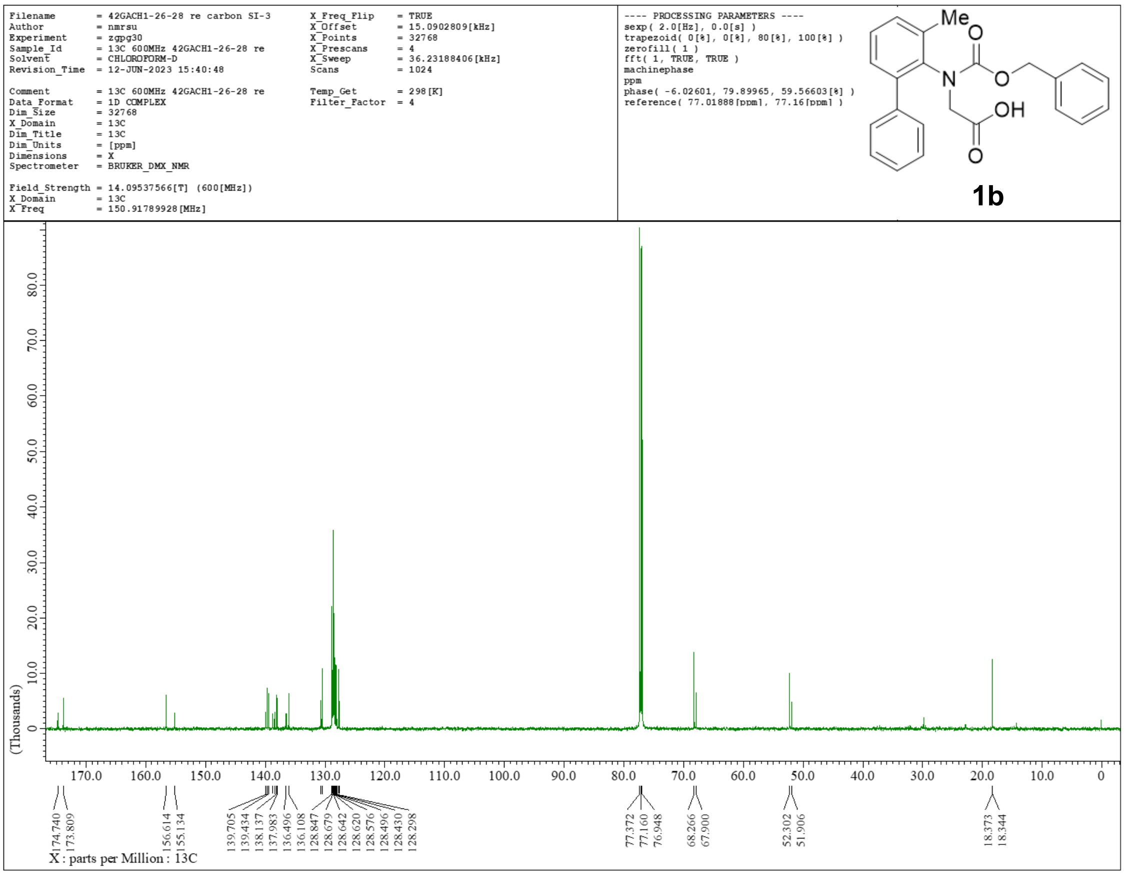

^{1}H NMR (600 MHz, $CDCl_3$, ppm) of **6**

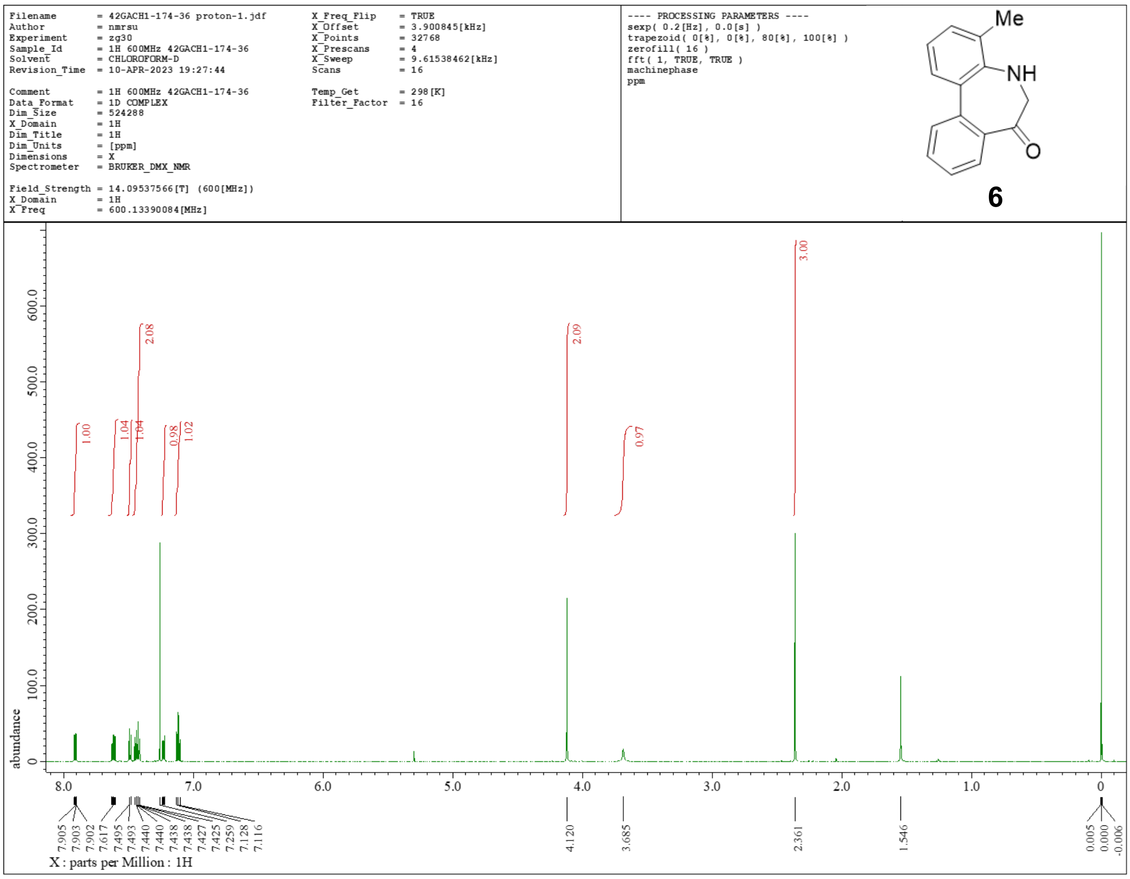

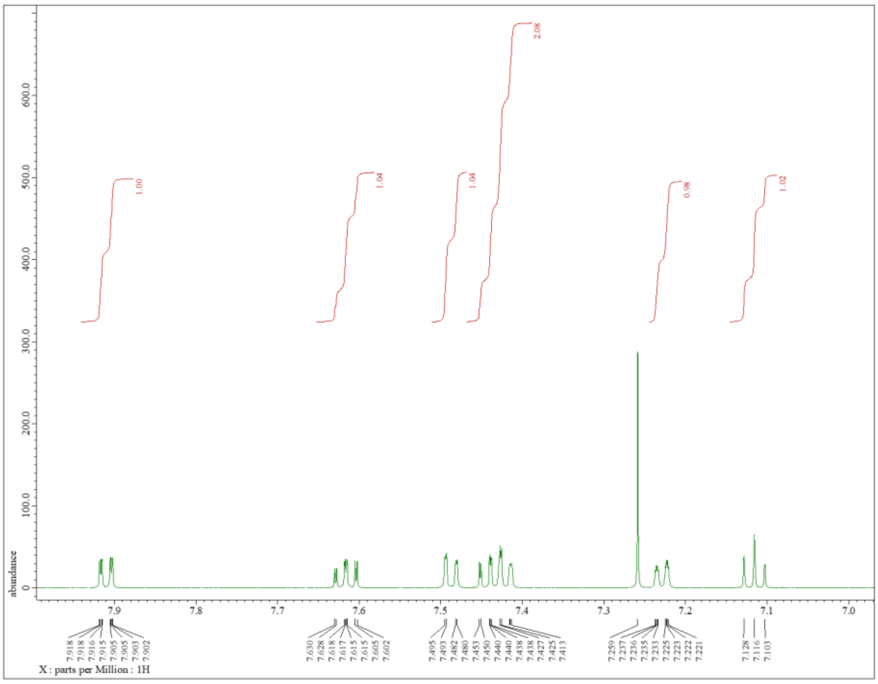

^{13}C NMR (150 MHz, $CDCl_3$, ppm) of **6**

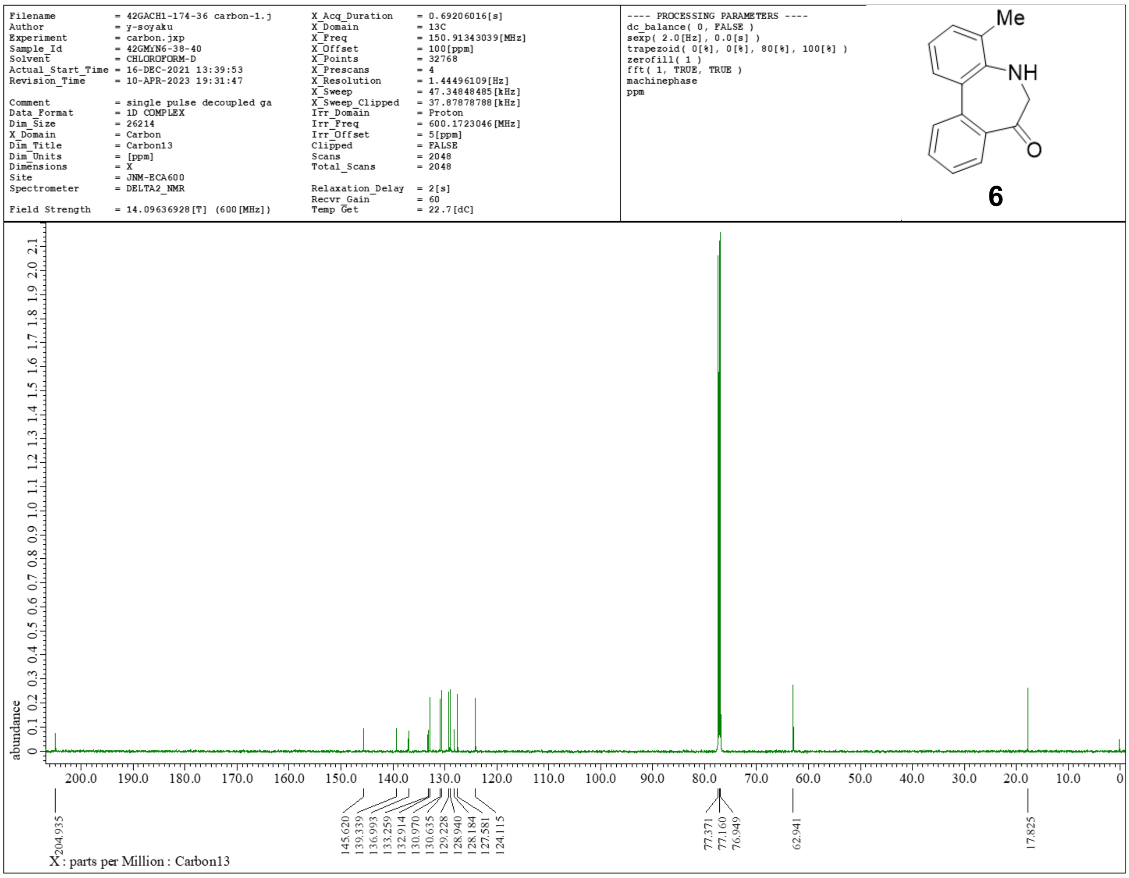

^{1}H NMR (600 MHz, $CDCl_3$, ppm) of **2a**

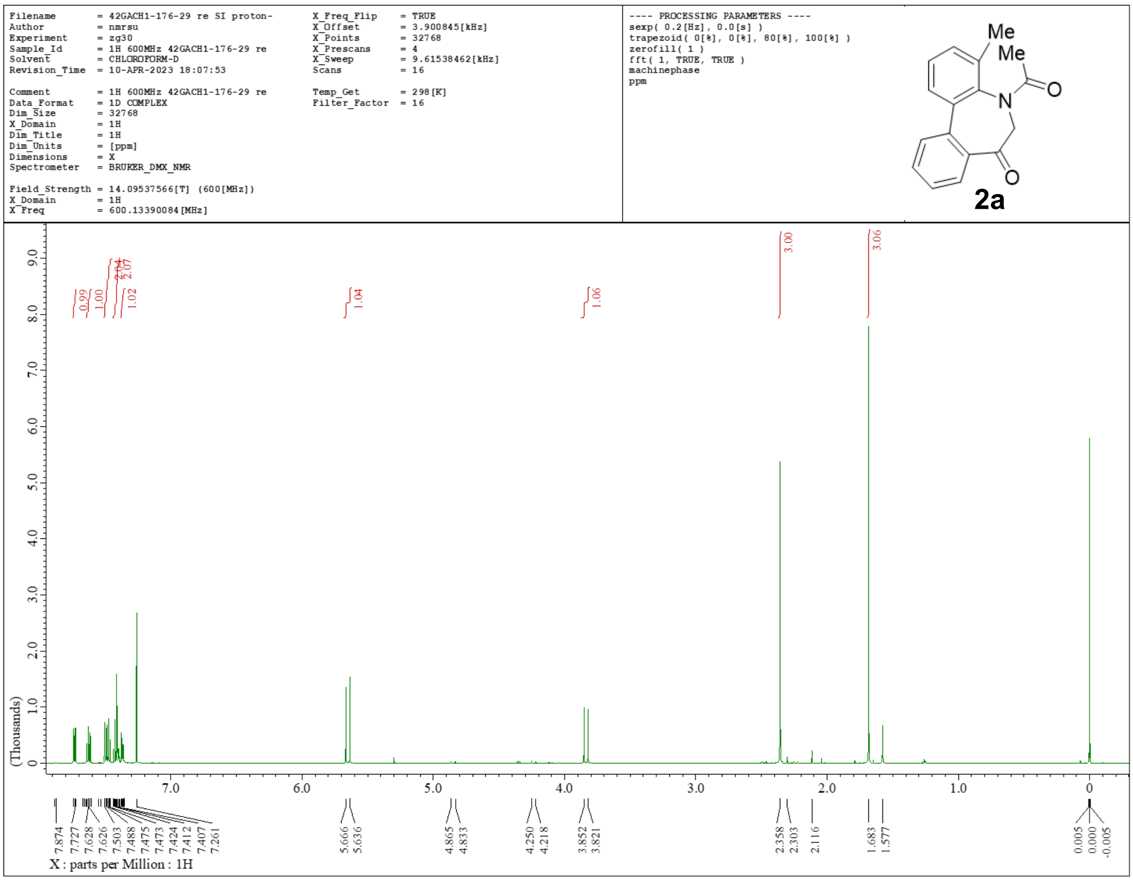

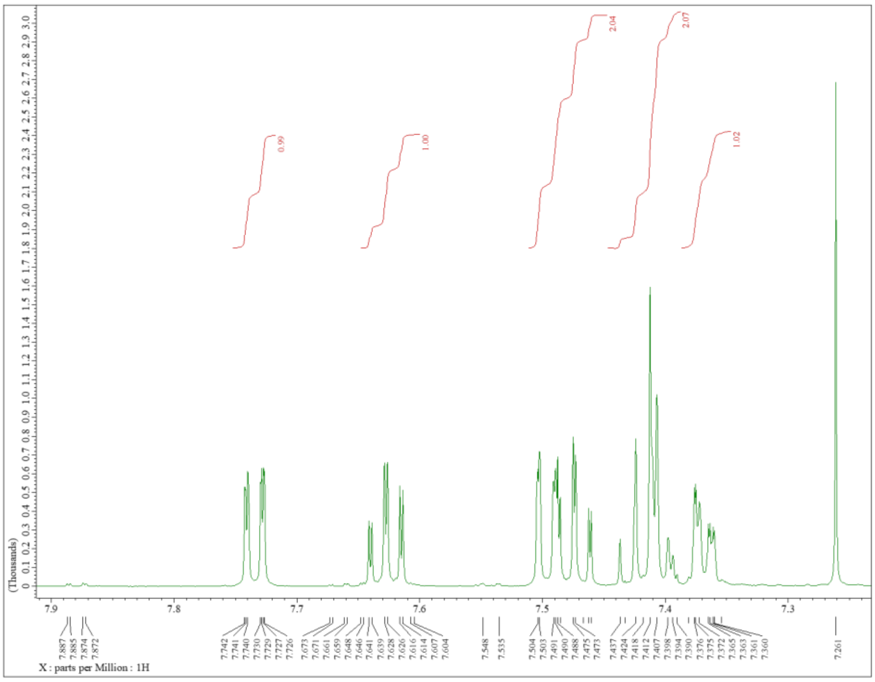

^{13}C NMR (150 MHz, $CDCl_3$, ppm) of **2a**

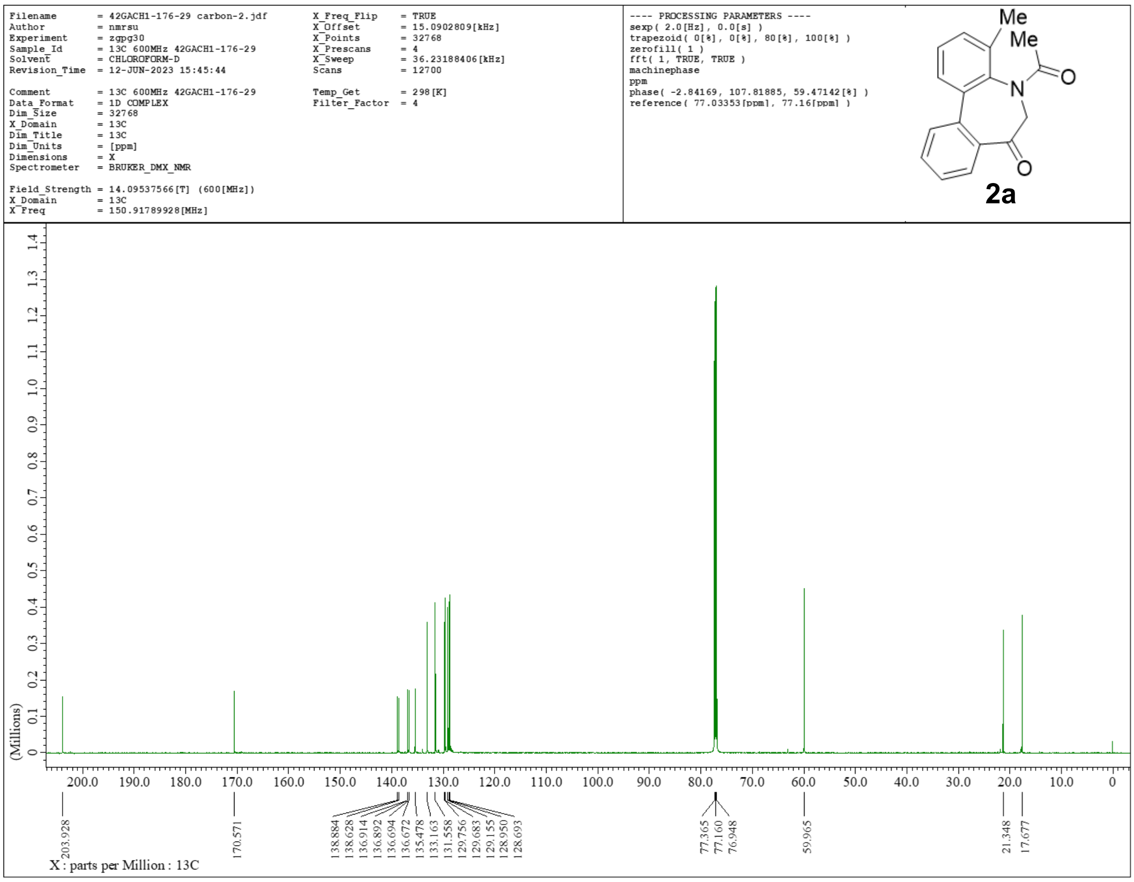

^{1}H NMR (600 MHz, $CDCl_3$, ppm) of **2b**

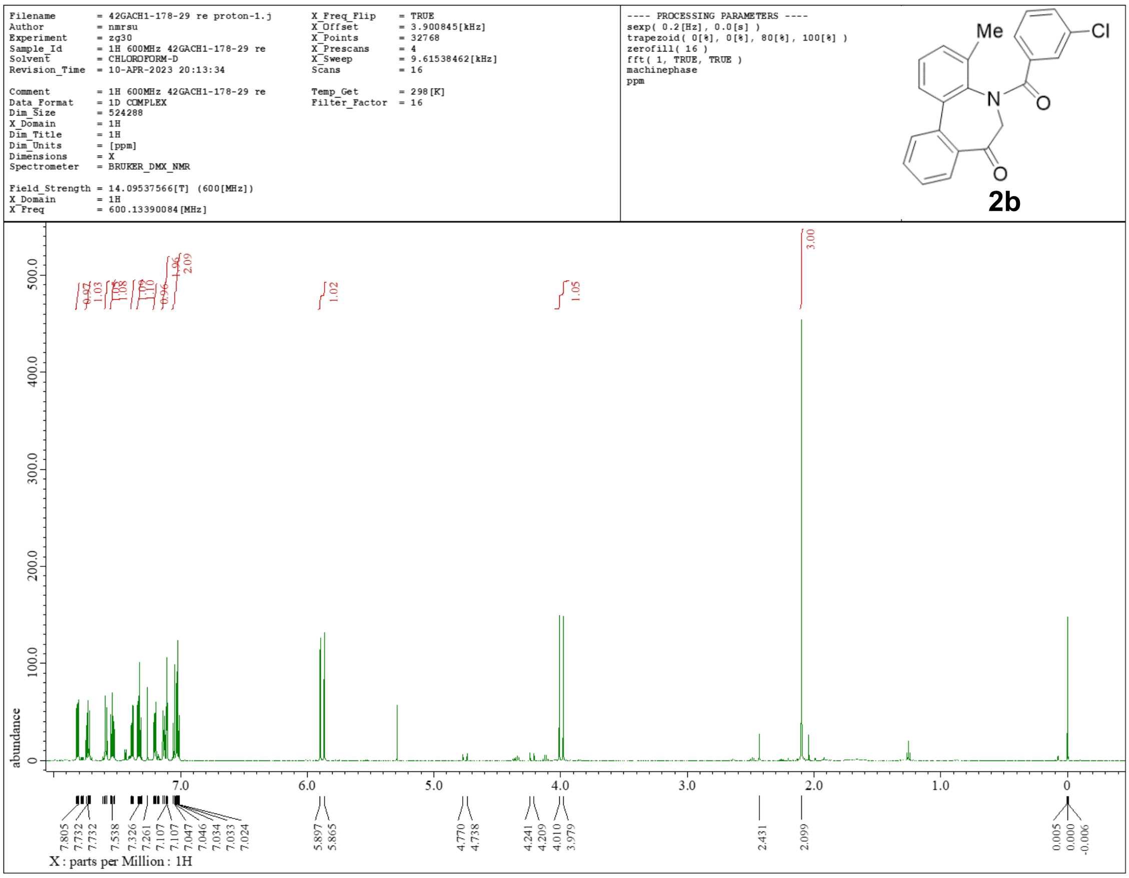

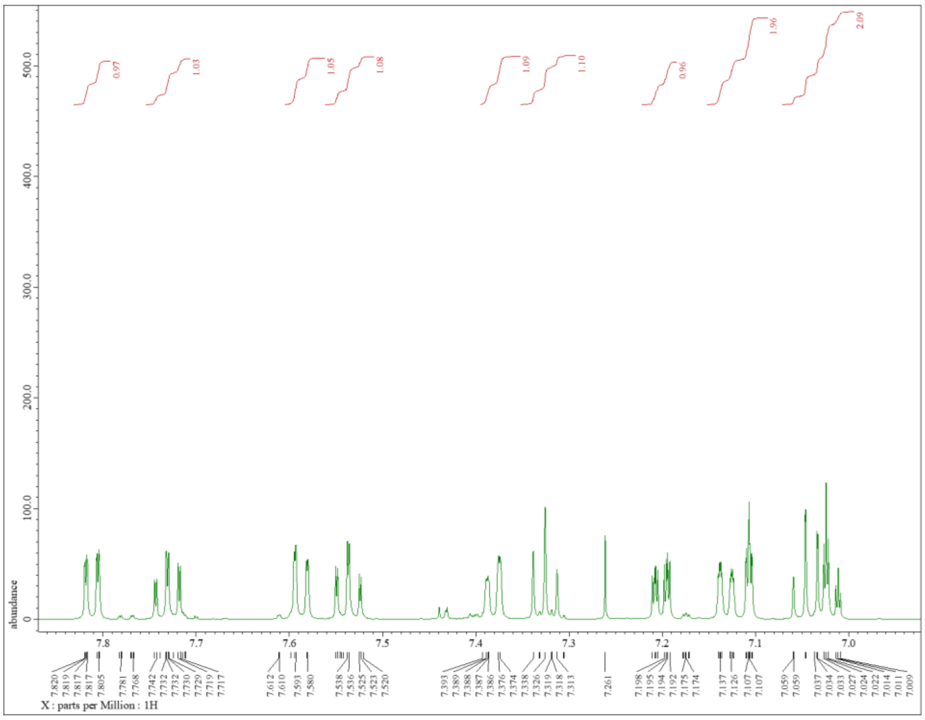

^{13}C NMR (150 MHz, $CDCl_3$, ppm) of **2b**

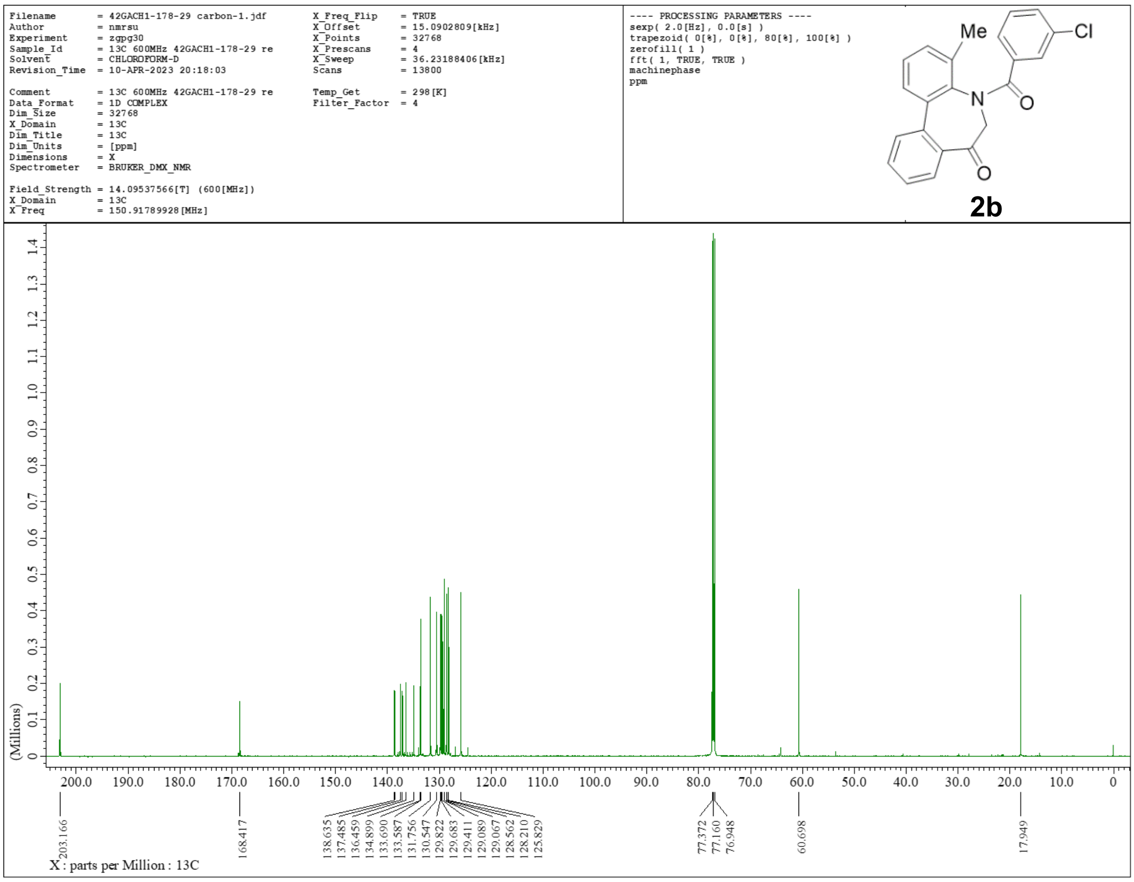

^{1}H NMR (600 MHz, $CDCl_3$, ppm) of **2c**

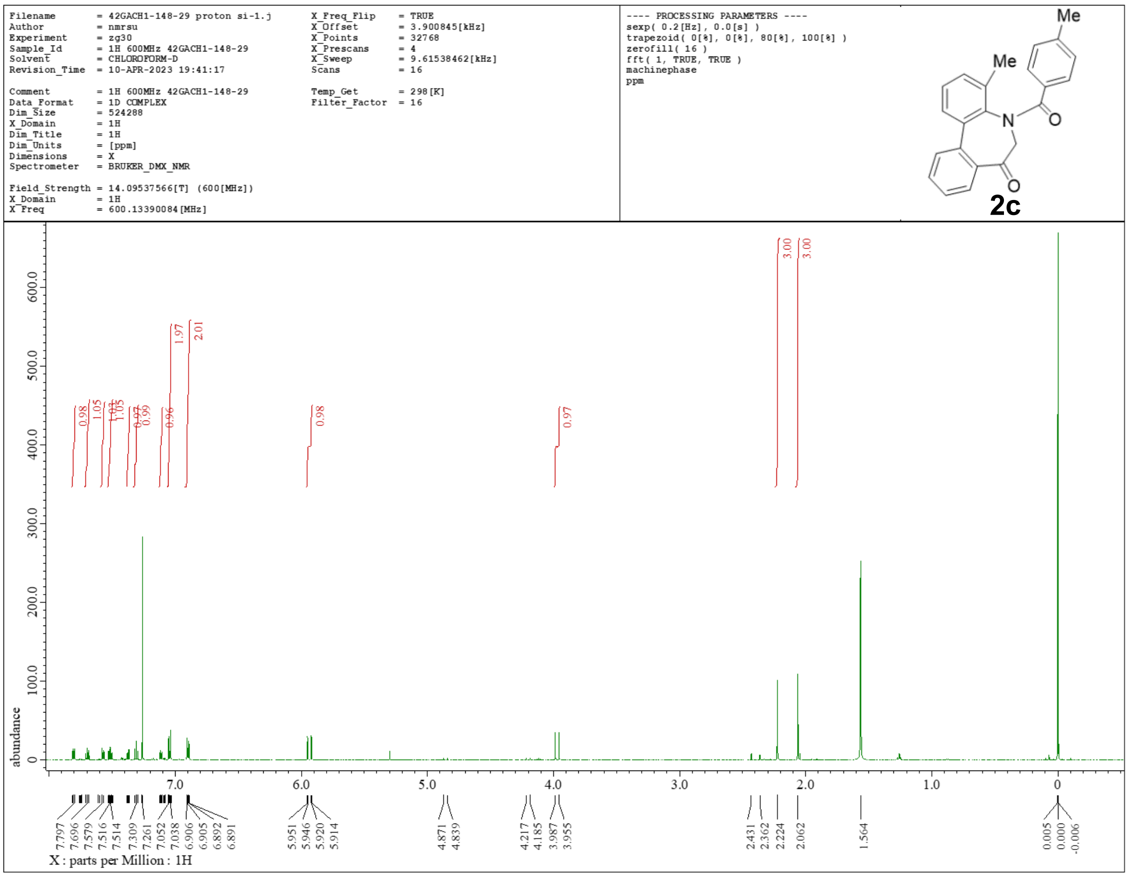

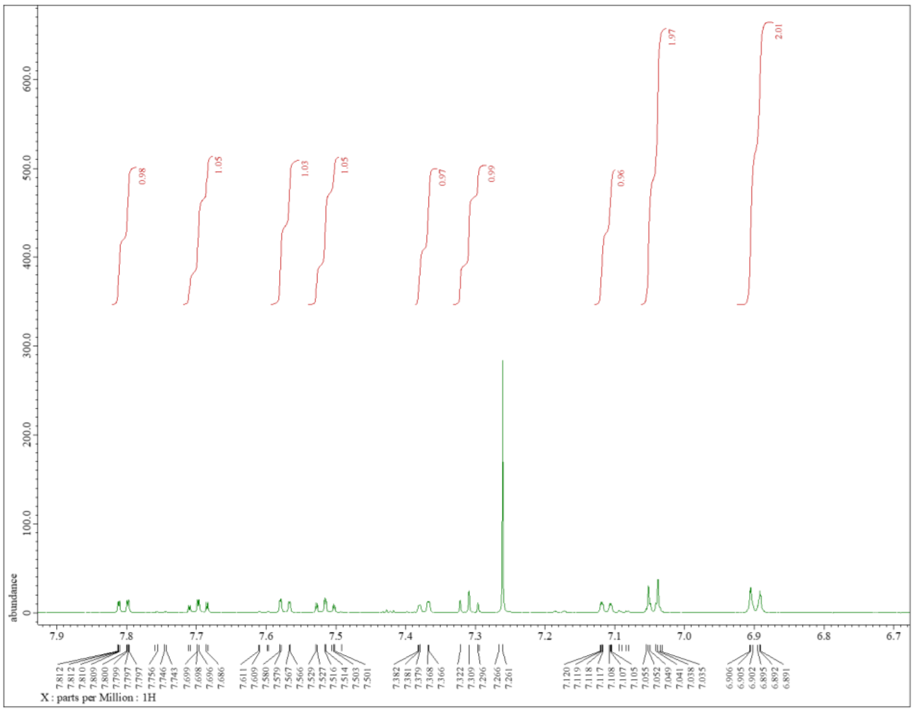

^{13}C NMR (150 MHz, $CDCl_3$, ppm) of **2c**

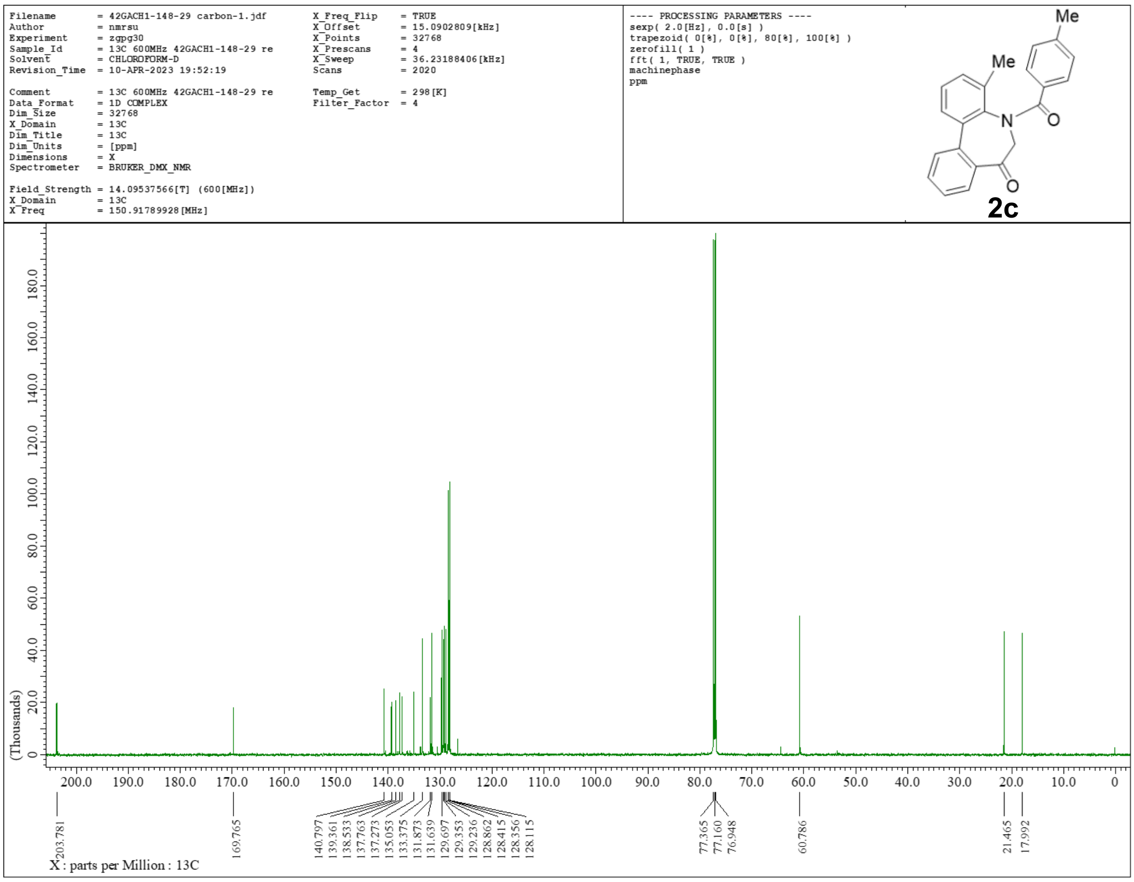

Supplement: Supplementary file 1 [file molecules-28-04734-s001.zip › molecules-2397011-supplementary.pdf]
